# Are you thinking what I’m thinking? Perspective-taking in a language game

**DOI:** 10.1371/journal.pone.0288330

**Published:** 2024-01-05

**Authors:** Johanne Nedergaard, Kenny Smith

**Affiliations:** 1 Department of Nordic Studies and Linguistics, University of Copenhagen, Copenhagen, Denmark; 2 School of Philosophy, Psychology and Language Sciences, University of Edinburgh, Edinburgh, United Kingdom; Wilfrid Laurier University, CANADA

## Abstract

Many theories of communication claim that perspective-taking is a fundamental component of the successful design of utterances for a specific audience. In three experiments, we investigated perspective-taking in a constrained communication situation: Participants played a word guessing game where each trial required them to select a clue word to communicate a single target word to their partner. In many cases, the task requires participants to take the perspective of their partner when generating, evaluating, and selecting potential clue words. For example, if the target word was ‘heart’, the first word that came to mind might be ‘love’, but this would not in fact be a very useful clue word. Instead, a word like ‘cardiovascular’ is much more likely than ‘love’ to make the partner guess ‘heart’. Pairs of participants took turns giving and receiving clues to guess target words, receiving feedback after each trial. In Experiment 1, participants appeared unable to improve their perspective-taking over repeated interactions, despite a baseline performance that suggested strong perspective-taking abilities. In Experiment 2, which included extensive feedback after each trial and only target words for which good clues existed and which required perspective-taking, some measures of perspective-taking showed modest improvements. In Experiment 3, which was conducted online, we used Experiment 2 feedback with Experiment 1 target words. As in Experiment 1, participants did not improve over the course of the game in Experiment 3. The results of these three experiments show quite strong limits on people’s ability to adapt and improve perspective-taking without the context provided by interaction history and growing common ground.

## 1. Introduction

Given the pervasive ambiguity of natural language, how do we know what to say and how it will be received by our audience? One of the most immediately relevant mechanisms underlying the design and interpretation of utterances is perspective-taking; what people do when they take the knowledge, beliefs, and lack thereof of their interlocutors into account, especially when these differ from their own. Considerable controversy remains surrounding both whether people are *able* to generate, simulate, manipulate, and maintain representations of other people’s perspectives (see e.g., [1, 2]) and whether people habitually *use* this ability in communication [3, 4]. In terms of whether perspective-taking is even feasible, several authors have argued that representing the perspectives of others is likely to be a highly cognitively demanding task, perhaps too demanding to deploy in time-pressured situations like conversation (a problem first identified in [5, 6]). Nevertheless, people seem to take into consideration what their interlocutor sees and knows at least some of the time (see e.g., [7]). In the current paper, we investigate putative cognitive mechanisms in perspective-taking: the generation of potential utterances and the evaluation of which potential utterances are most likely to lead to successful communication. We present three experiments that explore whether and how people are able to use perspective-taking in a constrained communication situation where perspective-taking involves modeling their interlocutor’s semantic associations, and whether participants can adapt over the course of many such communicative episodes, increasing their use of perspective-taking or its accuracy in order to increase success on the task. Through these experiments, we aim to shed light on important questions about the upper and lower bounds of people’s ability to simulate an interlocutor’s perspective.

### 1.1 Perspective-taking with visual grounding

Empirical studies investigating audience design come in a variety of methodological flavours, most of which rely on perspective-taking in communicative tasks involving a physically-present set of referents, which allows perspective-taking to be operationalized as how participants use physical visibility to speakers or hearers to guide their production or interpretation of utterances. Eye-tracking studies with adult participants during comprehension have indicated that hearers keep track of the speakers’ perspectives when interpreting their utterances [8–12]. For example, Heller et al. [12] tested what happened when speakers provided instructions for hearers to manipulate objects that were either visible to both participants or only to the hearer. If the hearer and the speaker could see different parts of the same visual scene, the hearer only looked at the objects that were plausible targets based on what the speaker could see. For example, if the speaker gave instructions to ‘pick up the big …’, the hearer would preemptively disregard a competing item (e.g., a big box contrasting with a smaller box) if the hearer knew that the speaker could only see one box. Presumably, this means that the hearer reasons that the speaker would not use the contrasting adjective (e.g., ‘big box’) if the speaker only had visual access to one item–instead, they would simply say ‘pick up the box’. While the above-mentioned studies have focused on adult communication, Nadig and Sedivy [13] also showed that even children as young as five years tailored their instructions to the information the hearer had access to. When the speaker’s task was to make the hearer manipulate an object that appeared in a size-contrasted pair, the speaker always used a contrastive adjective. However, when the hearer could not see one of the members of the pair, speakers only used the contrastive adjective half the time, indicating they took the hearer’s perspective into account in designing their utterance.

While these studies provide some empirical support for the idea that both speakers and hearers take perspective when designing and interpreting utterances, there are some important criticisms of the methodologies. For example, keeping track of other people’s knowledge of visually presented scenes (visual perspective-taking) could be less cognitively demanding than perspective-taking that is not visually grounded (conceptual perspective-taking): all participants have to do when designing and interpreting utterances in visually grounded cases is look at the scene in front of them; they are not required to model, maintain, update, and manipulate full-blown mental representations of other people’s worldview but can simply infer their perspective directly based on the visual scene (which might be a relatively low-cost cognitive operation, and possibly automatic, e.g., [14–16]). However, participants in such situations do frequently need to actively suppress visually salient information that is only accessible to them, which may count against the idea that visual perspective-taking is less demanding than conceptual perspective-taking. Nevertheless, the visually grounded approach risks neglecting many common real-life situations (e.g., talking about events occurring in a different location or at a different time, or talking about abstract concepts) where perspective-taking cannot be grounded in a visual scene with the necessary information directly available to the senses. In addition, the visually grounded experiments are sometimes designed in such a way that it is not possible to distinguish the effect of mutual knowledge from the effect of information known only to the individual (e.g., [17, 18] see [19] for a critical perspective). Importantly, this is not the case in all studies but it is nevertheless an essential experimental design feature to keep in mind. Clark, Schreuder, and Buttrick [17], for example, claimed to show an effect of common ground in interpreting demonstrative reference (e.g., ‘how would you describe the colour of *this flower*?’ referring to one particular perceptually salient flower in an array of flowers). Which flower appeared most salient was the same for both the hearer and the speaker, however, meaning hearers could just rely on their own perspective to interpret the demonstrative reference and did not have to use common ground information. It may be the case that most real-life interactions are like this [20], and so ‘directly computing what another person knows or does not know at a given moment might be more trouble than it is worth’ ([20], p. 39). Nevertheless, communication does not consistently fail in situations where people have differing perspectives (e.g., when an expert has to verbally instruct a novice) and so we do appear to have mental-state modelling mechanisms that we need to explain. Thus, it is important to investigate perspective-taking in situations where the interlocutors’ perspectives are different.

While the studies described above suggest both speakers and hearers use information about shared visual context adaptively at least sometimes, this does not always appear to be the case (with adult participants, [21]; or with children and adolescents, [22]). Introducing additional demands on mental resources increases egocentricity in perspective-taking. For example, Horton and Keysar [23] tested adult participants and found that time pressure prevented speakers from taking into account what they knew about the hearer’s perspective on the visual scene–instead, speakers provided instructions based on their own perspective. Zhao et al. [24] also found that memory demands and a bigger common ground size (i.e., more objects that both participants had access to) were associated with more egocentric errors for 8- and 10-year-olds. Similarly, and in contrast to the findings by Heller et al. [12] described above, Keysar, Barr, and Balin [25] found that adult hearers were equally likely to look at objects that were not part of the common ground and objects that were (see also [3]). Yet other authors have challenged the idea that participants’ apparent egocentric bias in visually grounded communication problems is indeed a failure of perspective-taking, instead arguing that it is caused by artificially high demands on selective attention [26] or artificially skewed allocation of perspective-taking resources between director and matcher ([27, 28]; henceforth we will term the ‘speaker’, i.e. the participant providing the clue/directions, ‘director’, and the ‘hearer’, i.e. the participant making the guess/selection, ‘matcher’). To come to a more complete understanding of these conflicting findings, it could be useful to examine the cognitive mechanisms underlying or comprising perspective-taking. One suggestion [21] is that perspective-taking can be decomposed into three subprocesses: 1) computing perspective information (what the interlocutor sees/knows/thinks), 2) holding perspective information in mind and remembering it for further communicative interactions, and 3) using perspective information (designing or interpreting utterances with perspective information in mind). These subprocesses fit the visual world paradigms discussed in the present section best as this is the context in which they were hypothesized. To evaluate how relevant they are to perspective-taking more generally, it is necessary to move beyond visual world paradigms.

### 1.2 Perspective-taking without visual grounding

As reviewed above, studies of communication based around partially-shared visual context show a mixed pattern of results, only some of which support perspective-taking in communication. What these studies have in common is that the conversation and context are quite rich with many opportunities over the course of an interaction to use interaction to align perspectives. A smaller parallel literature studies perspective-taking in much more impoverished contexts in an attempt to tap more directly into perspective-taking in communication. Sulik and Lupyan [29] tested perspective-taking in novel signalling tasks in a series of experiments where adult participants had to provide a clue word (e.g., ‘bulb’) to make their partner guess a target word (e.g., ‘light’). Participants did not switch roles during the study, i.e. the roles of director and matcher are fixed throughout. To achieve success on Sulik and Lupyan’s task, directors needed to provide a clue word that would strongly trigger the target word–thus, they had to consider possible clue words and possible matcher responses to those clues (i.e., how likely the matcher would be to select the target based on that clue) in order to identify effective clue words. This experimental method thus potentially lends itself more readily to illuminating mechanisms of real-life conceptual perspective-taking [20] which does not crucially depend on visual perspective. It is of course worth noting that this task is itself artificially constrained: for instance, people in real-world communication can use more than one word to communicate and can exploit a range of multimodal mutual feedback cues. Sulik and Lupyan manipulated whether speaker and hearer had *symmetric* or *asymmetric* perspectives in this task. A symmetric trial occurred if the first associate of the target word (the word with the highest elicitation probability given the target word) also had the target word as its first associate. For example, the first associate of ‘day’ is ‘night’ (i.e., most people when cued with ‘day’ will say ‘night’), and the first associate of ‘night’ is ‘day’ (i.e., most people cued with ‘night’ will say ‘day’); pairs could therefore succeed on symmetric trials by providing their own first associate as clue word and resulting guess. In other words, success on symmetric tasks does not strictly require perspective-taking by either director or matcher. In contrast, in asymmetric trials the use of first associates is unlikely to lead to success: for example, if the target word is ‘dolphin’, the first associate of ‘dolphin’ is ‘mammal’ but the first associate of ‘mammal’ is not ‘dolphin’ but ‘animal’. Only success on asymmetric trials provides unambiguous evidence of perspective-taking as this is the only case where the director using her own first association as a clue is highly unlikely to lead to a successful guess (e.g. if the target word is ‘dolphin’: the director selects their first associate ‘mammal’ as the clue word, the matcher selects their first associate ‘animal’ as their guess). The role that symmetry of associations plays in Sulik and Lupyan [29]–and in the experiments in our present study–is essential for the conclusions we can draw about perspective-taking. On critical asymmetric trials, the director must realize that they cannot succeed if they use what they know without taking into account the task faced by their interlocutor. On symmetric trials, the communication problem can be solved whether the director takes perspective or not, and we cannot know if they have done so as they would choose the same clue word regardless.

Participants in Sulik and Lupyan’s study generally failed to take perspective: Directors typically produced the clue most strongly associated with the target from their own perspective, rather than providing clues which would be likely to elicit the target from the matcher’s perspective. Sulik and Lupyan found that perspective-taking could be boosted by constraining the clue space, rather than giving directors an open-ended choice of clue words, by giving directors only a limited number of clues to choose from, or by giving both the director and matcher access to a limited number of potential target items. For example, if the target word was ‘bank’, directors were able to choose the most useful clue word if they only had to choose from the five potential clues ‘money’, ‘teller’, ‘vault’, ‘loan’, and ‘safe’ (constraining the clue space; the best option here is ‘teller’–at least for American English-speaking participants–because it most strongly cues ‘bank’). Similarly, directors were better at communicating target words when both they and the matchers had access to a small selection of potential targets. For example, if the target word was again ‘bank’, directors would be able to provide a useful clue word if their task was to select a clue to enable the matcher to select that target from among five items ‘bank’, ‘cash’, ‘fund’, ‘wallet’, and ‘profit’: it is tempting here to provide ‘money’ as a clue, but if the director takes the explicitly-provided potential targets into account, they should realize that ‘money’ would be a confusing clue word in this context.

To interpret these findings, it is necessary to consider the different cognitive mechanisms that could be involved in perspective-taking in this context. It seems that participants need to generate potential clue words, suppress their own egocentric perspective (ignore potential clue words that are salient from their own perspective but are unlikely to be successful, e.g., high-ranked associates of the target word which do not have the target word among their high-ranked associates), and evaluate the clue words by simulating their partner’s reaction. It appears that the participants in Sulik and Lupyan’s study found it especially difficult to generate potential clue words; evaluating which clue word would be the most effective when provided with a set of possibilities seemed to be easier.

Importantly, participants in Sulik and Lupyan’s study did not interact and were not told after each trial whether their clue or guess had been successful, nor what the matcher had guessed or what the target word was in the case of an incorrect trial, meaning that participants had little opportunity to adapt to their partner or the task over the course of the experiment. In real-life communication, people interact repeatedly over the course of one or more interactions with an interlocutor, and over a lifetime of interactions with multiple interlocutors, and get direct and indirect feedback about whether their attempts at communicating have been successful or not [30–32]; this opens up the possibility that growing experience with perspective-taking in day-to-day communicative tasks might allow speakers to become increasingly adept at adopting and adapting to the perspective of their interlocutors. In particular, participants may improve their ability to suppress their egocentric perspective [33, 34] when they experience that this does not lead to successful communication. One-shot instances of communication like these may show what kind of behavior comes first and easiest to people but to examine communication as a practised skill (which natural language use is), it is necessary to study repeated interactions. In a follow-up conference paper [35], Sulik and Lupyan in fact found that participants were able to improve their perspective-taking over repeated interactions in a face-to-face target-clue-guess task when they received feedback after each trial. However, the preliminary nature of [35] warrants further investigation of the role of interaction and feedback in perspective-taking tasks.

To contextualize the contribution of studies like these in terms of the visual world paradigms discussed in the previous section, it is useful to consider Apperly et al.’s proposal [21] of three subprocesses involved in perspective-taking (computing perspective information, holding perspective information in mind, and using perspective information). ‘Using perspective information’ can plausibly be mapped onto evaluating potential clue words (‘what would this word make my partner think of?’) while ‘computing perspective information’ parallels acknowledging that I (the director) have seen and now know what the target word is while you (the matcher) have not. Sulik and Lupyan’s paradigm leaves out the requirement to ‘keep perspective information in mind’ as the same target words do not reappear and focuses instead on ‘using perspective information’. This is an important contrast with the perspective-taking involved in real-life communication where we can also rely on the way words have previously been used in interactions. As for ‘using perspective information’, it appears that there are at least two components of this subprocess. The director both has to generate potential utterances and evaluate potential utterances. The generation of potential utterances presumably happens through forward association (the strongest associates come first to mind, and so on) while evaluating potential utterances potentially happens through backward association with the speaker asking themselves: ‘What kind of mental state would I put my conversational partner in if I used this utterance? Is it the one I intended?’ These processes are similar to processes found at the level of syntax where studies have for example examined whether speakers can evaluate their utterances for ambiguity and revise this ambiguity in light of what they think the listener knows [36, 37], and whether listeners use disfluencies in speech to make inferences about upcoming referents [38]. In our present study, we aim to shed further light on these mechanisms of generation and evaluation of potential utterances as well as answer important questions about what (if anything) can improve over the course of repeated interactions.

### 1.3 The present study

Because perspective-taking is often conceptualized as a core component of successful real-life communication, we aimed to test whether people could use perspective-taking adaptively when there were no other alternative means of achieving success. Particularly, if egocentric-first accounts are correct (e.g., [3, 4, 20, 25, 39]), then we might expect participants to learn to eliminate obvious potential clues (salient from their own perspective) from consideration and use cues that are better suited from the matcher’s perspective on the task. Our method was inspired by Sulik and Lupyan [29], featuring the same director-matcher task; in contrast to Sulik and Lupyan [29], the participants take turns being director and matcher and are provided with feedback (success, the target word, and the guess word) after each trial. In accordance with the preliminary findings from Sulik and Lupyan [35], we hypothesized that feedback would play an essential part in learning to improve perspective-taking. However, in contrast to Sulik and Lupyan [35], our participants interacted over a larger number of trials, took turns playing the roles of director and matcher (rather than having fixed roles for the duration of the experiment), and were not able to see each other. Importantly, target words never repeated. Repeated target words could lead to successful trials based on memory of previous successful interactions rather than active perspective-taking; by using each target word only once, any improvement over trials in our task would come from participants learning that using clue words that are salient from their own perspective (e.g., providing ‘mammal’ as a clue for ‘dolphin’) is not necessarily helpful and that they have to model allocentric semantic associations to achieve success. Improving perspective-taking in this context specifically means recognizing the importance of allocentric salience, and generating and evaluating clue words that are highly likely to elicit the target word from the matcher.

As we have seen above, symmetry (whether speaker’s and hearer’s perspectives actually differ) and salience appear to be important concepts when measuring perspective-taking in communication. We operationalized symmetry in a similar way to Sulik and Lupyan [29], quantifying egocentric and allocentric salience (i.e., salience from one’s own or one’s partner’s perspective) by word association strength, a measure of how strongly a word cues another word (see Table 1).

**Table 1:** Overview of the different measures of association strength as conditional probabilities between targets, clues and guesses. Forward association strength for the director for example denotes the conditional probability of the clue word given the target word. This operationalizes egocentric salience because it concerns the first words that come to mind when the director sees the target word, if the director does not take into account that they have to select a clue word that is useful for the matcher. Forward association strength therefore operationalizes egocentric salience while backward association strength operationalizes allocentric salience.

Association strength direction	Director	Matcher
***Forward (egocentric)***	p(clue|target)	p(guess|clue)
***Backward (allocentric)***	p(target|clue)	p(clue|guess)

Of all the measures of perspective-taking, **forward association strength** is the most straightforward: It denotes the probability of a response given a cue. In the case of the director, this would be the probability of the clue given the target (how likely is the target ‘whale’ to elicit the clue ‘ocean’?) while for the matcher, forward association strength would be the probability of the guess given the clue (how likely is the clue ‘ocean’ to elicit the guess ‘water’?). Because these are simple cue-response contingencies, they do not provide evidence of perspective-taking. Instead, we consider **backward association strength** the main measure of interest in this study. For the director, backward association strength denotes the probability of the target given the clue (how likely is the clue ‘ocean’ to lead the matcher to guess the target ‘whale’?) while for the matcher, backward association strength would be the probability of the clue given the guess, i.e., the matcher’s idea of the target word (how likely is the guess/potential target ‘water’ to have elicited the clue ‘ocean’?). In either case, the higher the backward association strength, the more evidence of perspective-taking.

**Fig 1:**
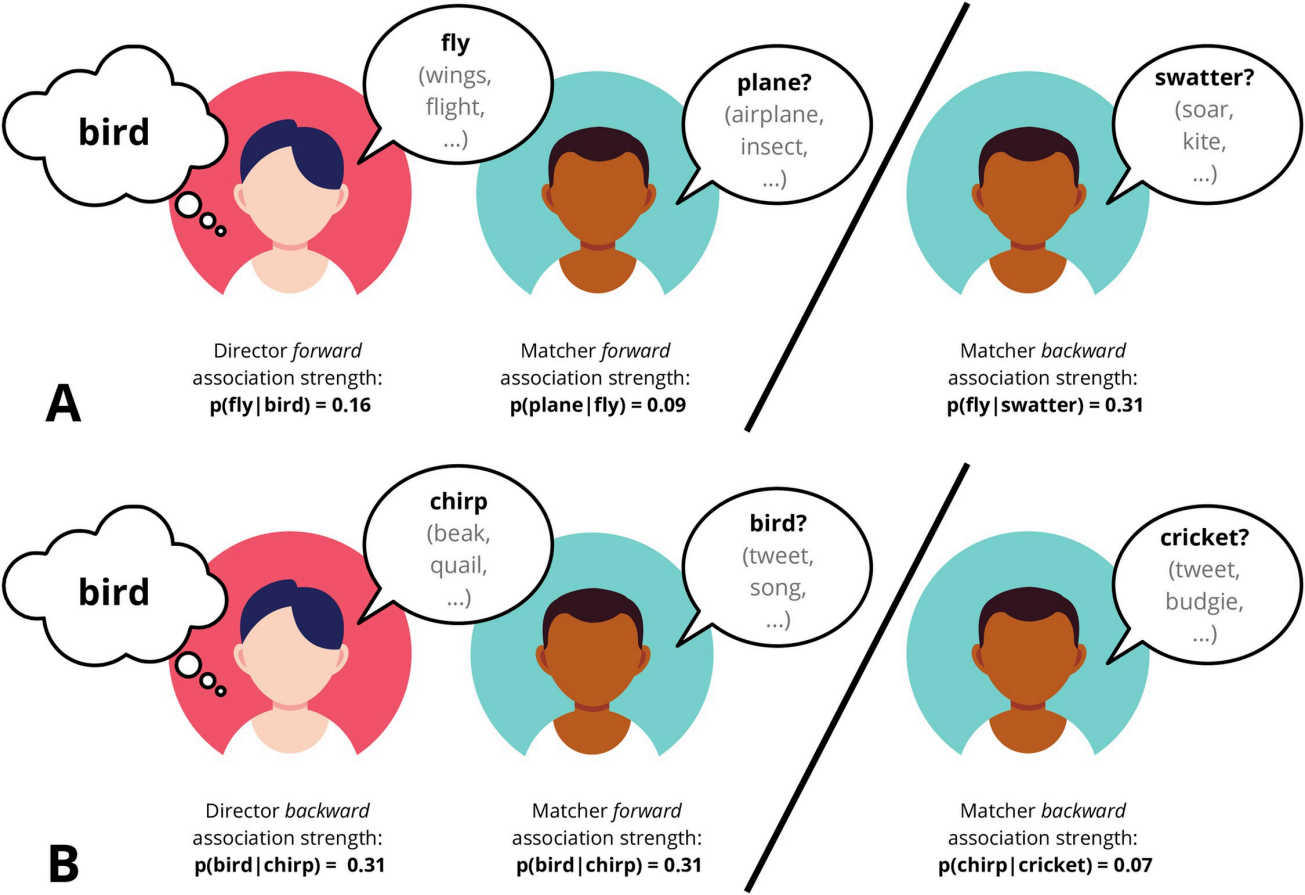
Schematic representation of how our measures of association strength played out in the experiments. In Panel A, the director is given the target word ‘bird’ and chooses the clue word based on highest forward association strength, in this case ‘fly’. The matcher also uses forward association strength to make their guess and guesses the highest ranked term ‘plane’. If the matcher had been using backward association strength to make their guess, they would have said ‘swatter’ (because ‘swatter’ is the most likely target word to have elicited the clue word ‘fly’). In Panel B, the director chooses their clue word based on the highest backward association strength which leads the matcher to correctly guess ‘bird’. If the matcher attempts to use backward association strength to infer what prompted the director to say ‘chirp’, they incorrectly guess ‘cricket’.

To illustrate what perspective-taking would look like in the present setup, consider the target word ‘plague’ (association strength measures in the following examples are computed from the large-scale word association study The Small World of Words [40]). A director relying solely on egocentric forward association strength would give ‘death’ as their clue, the highest ranked forward associate of ‘plague’ (p(death|plague) = 0.13). A director reasoning about allocentric salience, salience for the matcher, would choose ‘bubonic’ as their clue, which has the highest backward association strength with ‘plague’ (p(plague|bubonic) = 0.38). This means that giving ‘bubonic’ as a clue is more likely than alternatives (e.g., ‘death’) to lead the matcher to correctly guess the target word. See also Fig 1 for a schematic representation of a worked example. Thus, if the directors’ perspective-taking improved over rounds, we expect backward association strength to increase and forward association strength to decrease. Note that there is a potential additional challenge in this task of the division of labor in perspective-taking between director and matcher; in our calculation of backward association probabilities, we assume that both partners are *not* taking perspective (i.e., that they are selecting clues and guesses based on forward association strength rather than backward association strength). This potential for infinite regress in reasoning about interaction partners is one of the potential challenges of audience design; in the case of the director, we adopt the same resolution as in some computational models, e.g., the Rational Speech Act model [41], and assume that inferences about one’s partner bottom out in a model of a simple interlocutor, i.e., one who relies on forward associations. Under this assumption about the director’s behaviour, the matcher should assume that the director tried to provide as good a clue word as they could, freeing the matcher to rely on their own forward associations. However, we include the matcher’s backward associations as a dependent measure as it may nonetheless index attempts at perspective-taking by the matcher (e.g., if they realize that the director is not providing good clues).

We report results from 3 experiments which differ on the set of target words (both symmetric and asymmetric target words in Experiment 1 and 3 and only asymmetric target words in Experiment 2) and on the level of feedback provided to participants (only target, guess, and whether the guess was correct or incorrect in Experiment 1, the same in Experiments 2 and 3 with the addition of ‘from [clue] most people guess: [top associate]’ and ‘an even better clue would have been [highest ranking backward associate]).

## 2. Experiment 1

Participants played a word guessing game in pairs; on each trial, one participant (the director) was required to help their partner (the matcher) guess a single target word by providing a single clue word, with the roles of director and matcher alternating each trial.

### 2.1 Data availability statement

All stimulus materials and all data for the three experiments presented here can be found online at https://osf.io/ne9zy/.

### 2.2 Method

#### Participants

We recruited 40 participants (10 male and 30 female, mean age = 23.54, range = 18–32) playing as 20 dyads. The participants were recruited from the student population at the University of Edinburgh. All participants were self-reported native English speakers above the age of 18 and received £10 for their participation. The participants in pairs did not know each other before participating in the experiment, and the allocation to dyads was based on which timeslot participants signed up for (i.e. participants who signed up for the same timeslot formed a dyad). There were 14 same-gender dyads and 6 different-gender dyads, and the difference in age within a dyad ranged from 0 to 12 years (median = 2 years). The full study (all three experiments) received ethical approval from the PPLS Research Ethics Committee at the University of Edinburgh (protocol number: 296-1819/1). Participants in all 3 experiments provided informed consent, consent was given in writing (Experiments 1–2) or by mouse click (Experiment 3).

#### Materials

We selected 120 target words from the most common English nouns (using the iWeb Corpus; [42]) to ensure familiarity with meaning and spelling. We selected target words such that half the words in each list had top 1 or top 3 symmetric associates, and the other half had asymmetric associates. For example, the target word ‘term’ is top 1 symmetric because its top associate is ‘semester’, and the top associate of ‘semester’ is ‘term’; the target word ‘vehicle’ is top 3 symmetric because one of its top 3 associates (‘car’) has ‘vehicle’ as one of its top 3 associates. In contrast, ‘project’ is an asymmetric target word because none of its top 3 associates ‘work’, ‘task’, and ‘school’) in turn cue ‘project’ as one of their top 3 associates. All association strength measures came from the large-scale word association study The Small World of Words (SWOW; [40]). Sulik and Lupyan [29] compared results using the SWOW with results using the University of South Florida (USF) Free Association Norms [43] and the Edinburgh Associative Thesaurus (EAT; [44]) but found that different association norms produced similar results. Therefore, we only used the SWOW as it was the more extensive database. The target words were divided into six groups of 20 in which each group contained half symmetric and half asymmetric target words–these six groups comprised six rounds of the experiment in order to ensure that symmetric and asymmetric target words were evenly distributed throughout.

#### Procedure

**Fig 2:**
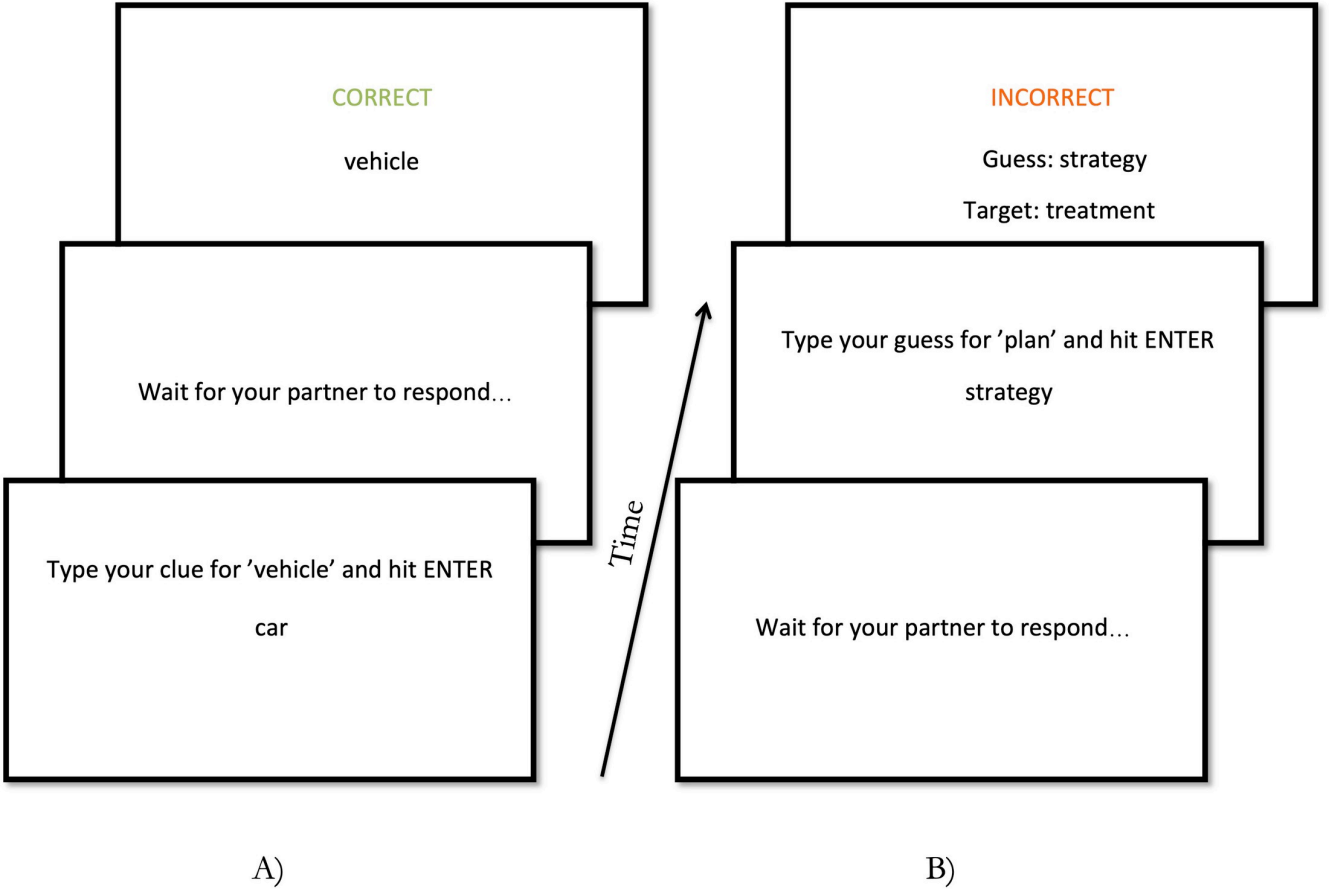
(A) a successful director trial: the director provides the clue ‘car’ for the target ‘vehicle’, and the matcher successfully guesses ‘vehicle’. (B) an unsuccessful matcher trial: the matcher guesses ‘strategy’ from the clue ‘plan’, but the target was actually ‘treatment’.

Participants were told they would be playing a word guessing game where the aim was to help their partner guess the target word using only one clue word. In addition to the on-screen instructions (Verbatim: ‘You are about to participate in a study which involves playing a language game with a partner. You will take turns sending clue words to your partner to guess a target word, and making guesses based on your partner’s clue word. The game is computerized, you will see the target words and the clue words on the screen in front of you, and your typed responses will be sent to your partner’s computer.’), participants were orally instructed by the experimenter to only use single words that exist in English and were given examples of trials demonstrating successful and unsuccessful perspective-taking (spoken words to the effect of “if you get the target word ‘whale’ and want to get your partner to guess it, it might not be very helpful for them to say ‘ocean’ because it is like to make them think ‘water’. Instead, something like ‘harpoon’ or ‘blubber’ is more likely to make your partner guess ‘whale”‘). Participants were seated in separate booths and communicated over networked computers using custom-written software in PsychoPy [45]. Participants took turns sending and receiving clues and both received feedback after each trial, being given the target, the guess, and whether the guess was correct or incorrect (see Fig 2 for example trials in Experiment 1).

The director on a given trial could type in any real English clue word (except one identical to the target word) and there was no time limit on giving a response. If the clue word was identical to the target word or the program detected a whitespace in the clue, the participant would be asked to try again. An experimenter monitored the game from a different cubicle as it went on and intervened if participants started using other disallowed strategies (e.g., mirror writing or partial writing of the target, for example ‘indu’ as a clue for ‘industry’). In practice, this was rare. The matcher received the clue word and typed in their guess, again without a time limit. Dyads had 20 trials in each round and played six rounds; the entire experiment lasted approximately one hour.

### 2.3 Results

If participants are able to perspective-take adaptively, we expected both success rate (proportion of correct guesses by the matcher) and perspective-taking (as measured by director and/or matcher backward association strength, with higher values suggesting better perspective-taking) to increase over the course of the game. The full set of dependent variables included the binary success outcome (match between target and matcher’s guess), director and matcher backward association strength, director and matcher forward association strength, clue rank (among all potential clue words in the SWOW corpus, how high was the rank of the clue word given?), guess rank (among all potential responses to the clue word in the SWOW corpus, how high was the rank of the guess?), and association strength ratio between the optimal clue and the clue given.

To better assess which properties of the target words influenced success rate, we also examined the independent variables *accessibility* (1^st^, 2^nd^, 3^rd^ and 4^th^ quartiles of director backward association strength of optimal clue word) and *symmetry* (top 1 symmetric, top 3 symmetric, and asymmetric). Note that accessibility does not refer to how easy it is for the director to generate clue words but rather how potentially accessible the target word is for the matcher. The accessibility variable operationalized the existence of ‘good’ clue words, i.e., words that strongly and specifically cued a given target. For example, the target word ‘eye’ had a good potential clue word in ‘retina’ (p(eye|retina) = 0.33) whereas the target word ‘department’ did not have a particularly good potential clue, the best one being ‘bureau’ (p(department|bureau) = 0.02). ‘Eye’ therefore falls in the 4^th^ quartile of accessibility (it should be relatively accessible, in that a good clue word does exist) whereas ‘department’ falls in the 1^st^ quartile of accessibility (even its best clue word has very low backwards association strength). In Experiment 1, the maximal director backward association strength (backward association strength between target and the optimal clue word) had an overall mean of 0.16 and ranged from 0.01 to 0.33; the 1^st^ quartile was 0.09 and the 3^rd^ quartile was 0.23.

#### Data cleaning and preparation

Three dyads ran out of time and did not complete all six rounds: One dyad only played three rounds, and two dyads only played four rounds. Their trials were still included in the analyses. Guess words with spelling mistakes, typos, plurals, and other standard spellings counted as correct in all three experiments (manually scored at the analysis stage). As participants could type any word for both clues and guesses, some of the clues and guesses did not appear in the SWOW norms and we were therefore unable to score association strengths. This was the case for 387 of the trials for director forward association strength (17.12%), 516 of the trials for matcher forward association strength (22.83%), 426 of the trials for director backward association strength, clue rank, and ratio between optimal clue and the clue given (18.85%), and 437 of the trials for matcher backward association strength and guess rank (19.34%). The proportions of associations that could not be looked up was relatively stable over the experiment, i.e. it was not the case that early or late rounds featured disproportionately many trials where we could not score association strengths. There were only a few cases where participants used disallowed words, and those only occurred in the beginning of the experiment. In those cases, the experimenter interrupted the session, repeated the instructions, and restarted the experiment from the beginning.

#### Descriptive statistics

**Fig 3:**
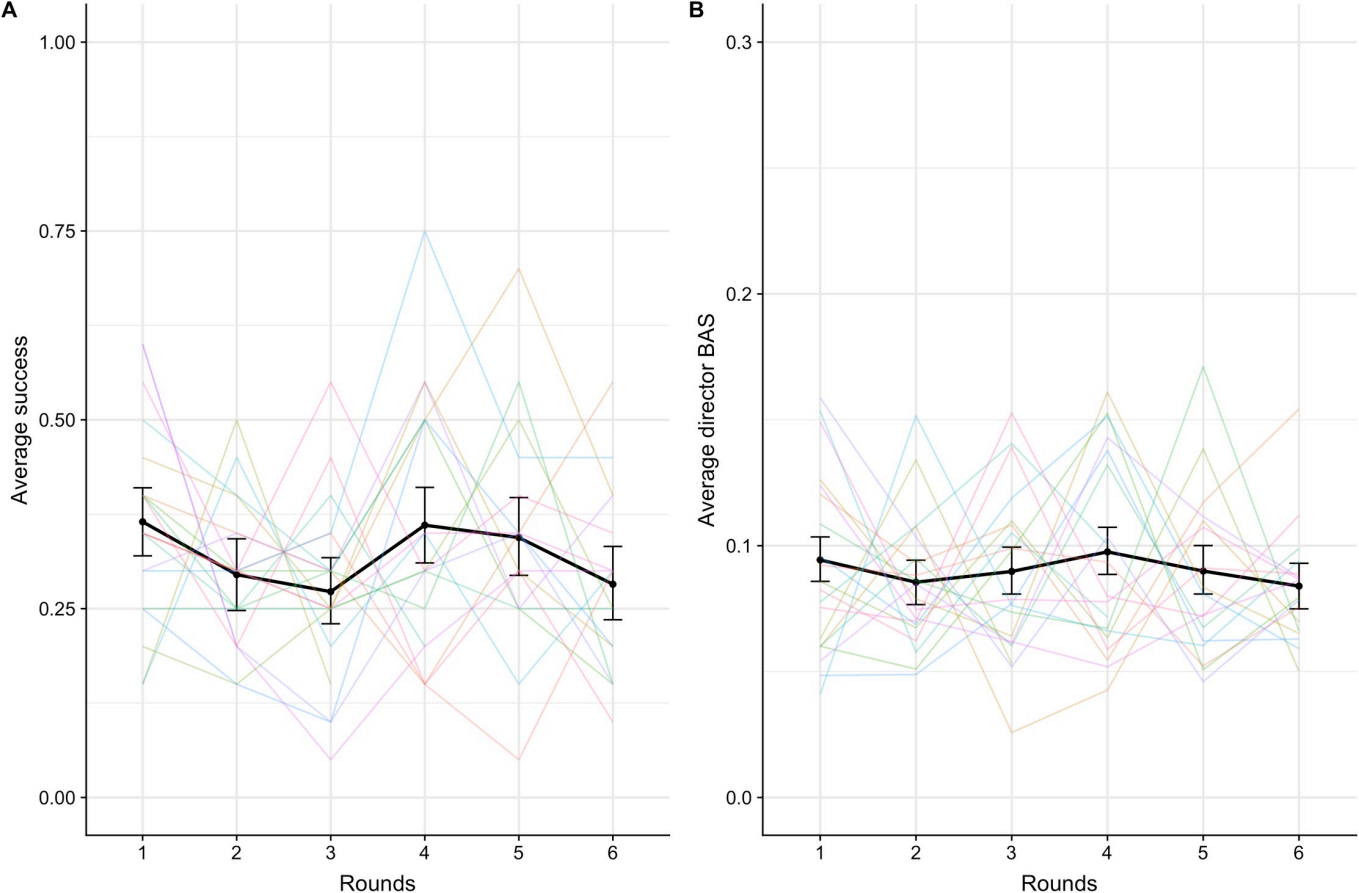
Success (A) and director backward association strength (B) over rounds. The black lines indicate averages across dyads with error bars showing bootstrapped 95% confidence intervals. Colored lines represent individual dyads. Note the different scales in A and B.

See Fig 3A for average success over rounds and Fig 3B for average director backward association strength over rounds. See Table 2 for the numerical descriptive statistics of all the dependent variables.

**Table 2:** Means and standard deviations (in parentheses) of success, RT (reaction time in seconds), director backward association strength (BAS), director forward association strength (FAS), matcher backward association strength (BAS), matcher forward association strength (FAS), clue rank, guess rank, and optimal:given (OG) ratio.

Rounds	Success	RT	Director BAS	Director FAS	Matcher BAS	Matcher FAS	Clue rank	Guess rank	OG ratio
1	0.37 (*0*.*48*)	32.00 (*20*.*80*)	0.094 (*0*.*08*)	0.06 (*0*.*06*)	0.11 (*0*.*09*)	0.07(*0*.*07*)	27.23 (*81*.*2*)	8.48 (*17*.*31*)	5.55 (*9*.*13*)
2	0.30 (*0*.*46*)	30.96 (*22*.*28*)	0.086 (*0*.*08*)	0.06 (*0*.*07*)	0.08 (*0*.*08*)	0.07 (*0*.*07*)	28.97 (*86*.*63*)	11.63 (*22*.*14*)	6.29 (*10*.*91*)
3	0.27 (*0*.*45*)	27.27 (*15*.*33*)	0.090 (*0*.*08*)	0.05 (*0*.*06*)	0.09 (*0*.*08*)	0.06 (*0*.*07*)	27.10 (*58*.*95*)	11.24 (*22*.*22*)	5.68 (*9*.*55*)
4	0.36 (*0*.*48*)	24.35 (*16*.*66*)	0.098 (*0*.*08*)	0.07 (*0*.*07*)	0.10 (*0*.*08*)	0.08 (*0*.*08*)	26.97 (*80*.*46*)	8.62 (*18*.*74*)	5.60 (*9*.*72*)
5	0.34 (*0*.*48*)	24.11 (*14*.*15*)	0.090 (*0*.*08*)	0.06 (*0*.*06*)	0.09 (*0*.*08*)	0.07(*0*.*07*)	31.24 (*70*.*79*)	8.06 (*16*.*53*)	6.08 (*9*.*58*)
6	0.28 (*0*.*45*)	22.22 (*11*.*95*)	0.084 (*0*.*08*)	0.05 (*0*.*06*)	0.09 (*0*.*08*)	0.07 (0.06)	30.58 (*57*.*25*)	8.10 (*15*.*37*)	6.15 (*10*.*31*)

#### Success

We adopted the following general procedure to fitting models: We attempted to model the maximal structure suitable for the experimental design, i.e. random intercepts and random slopes for target words and participants or dyads (depending on the dependent variable). For dependent variables that were determined by the dyad (like success), we used dyad for the random effects; for the dependent variables that were determined by individual participant (like forward and backward association strength, etc.), we used participant for the random effects. If the model then failed to converge or produced singular fit warnings, we set the slopes and intercepts to be uncorrelated. If the convergence problems remained, we excluded random effects based on conceptual reasoning (i.e. for predicting success across rounds, we wanted to control for the slope of the dyads so prioritized keeping the random slope over intercept for dyads). In the text, we report the most complex model that we were able to fit. We analyzed success predicted by round number with a binomial mixed-effects regression model with round as a fixed effect, by-dyad random intercept and (uncorrelated) random slope for round, and by-target word random intercept and random slope for round. This model indicated no effect of round on success (β = -0.01, SE = 0.04, p = .711).

**Fig 4:**
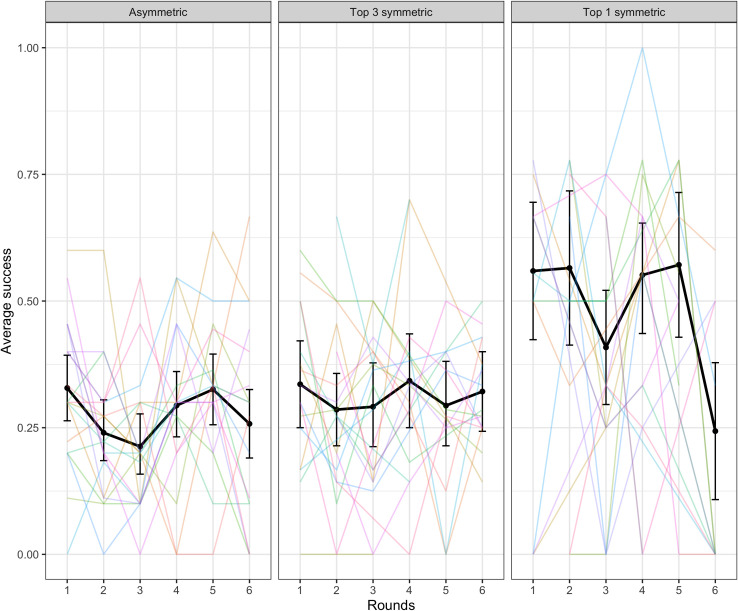
Success in the word guessing game by symmetry of the target word. The black lines indicate average across dyads with error bars showing bootstrapped 95% confidence intervals. Colored lines represent individual dyads. The curious pattern for Top 1 symmetric items in round six appears to be an artefact of the few observations (N = 37) in that data cell.

To verify that target words that required perspective-taking were harder, we ran a binomial mixed-effects regression model with success predicted by symmetry. The random effects structure for this model included random intercepts and slopes across rounds by target word and by-dyad random slopes across rounds. This model indicated that top 1 symmetric target words were significantly easier to communicate than asymmetric targets words (β = 1.10, SE = 0.29, p < .001). Top 3 symmetric targets were not significantly easier than asymmetric targets (p = .202). See Fig 4.

**Fig 5:**
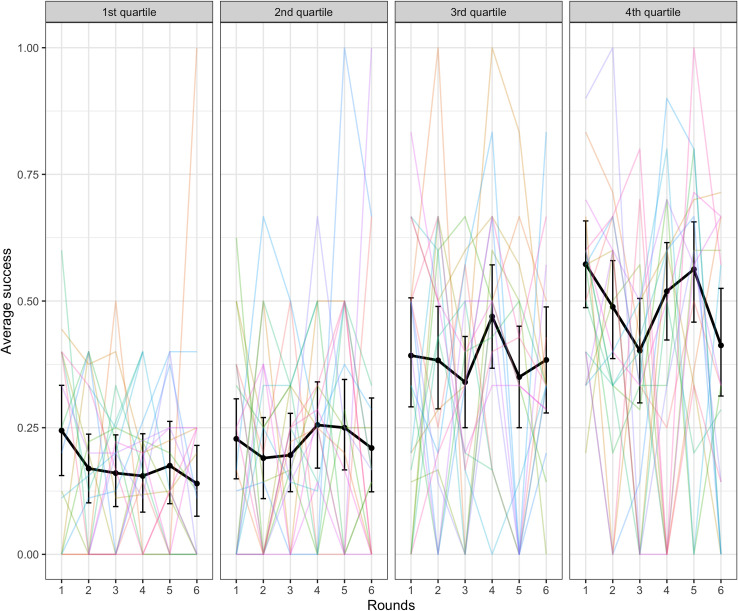
Success in the word guessing game by accessibility of the target word. The black lines indicate average across dyads with error bars showing bootstrapped 95% confidence intervals. Colored lines represent individual dyads.

We also tested the effect of accessibility on success, using a model with a fixed effect of accessibility comparing the 2^nd^, 3^rd^, and 4^th^ quantiles with the 1^st^ quantile as a baseline (the random effects structure for this binomial mixed-effects model consisted of by-dyad and by-target word random slopes over rounds and uncorrelated random intercepts for dyad and target word). Targets with accessibility scores in the 3rd and 4th quartiles (i.e., which had good clues available according to backwards association strength) were easier to communicate than the baseline 1^st^ quartile (1^st^ vs 2^nd^: β = 0.37, SE = 0.25, p = .136; 1^st^ vs 3^rd^: β = 1.26, SE = 0.24, p < .001; 1^st^ vs 4^th^: β = 1.84, SE = 0.24, p < .001). See Fig 5 below.

#### Director backward association strength

A linear mixed-effects regression model with by-director random slope for round and by-target word random intercept and random slope for round indicated no effect of round on director backward association strength (β = -0.01, SE = 0.01, p = .207).

#### Further measures of improvement

**Fig 6:**
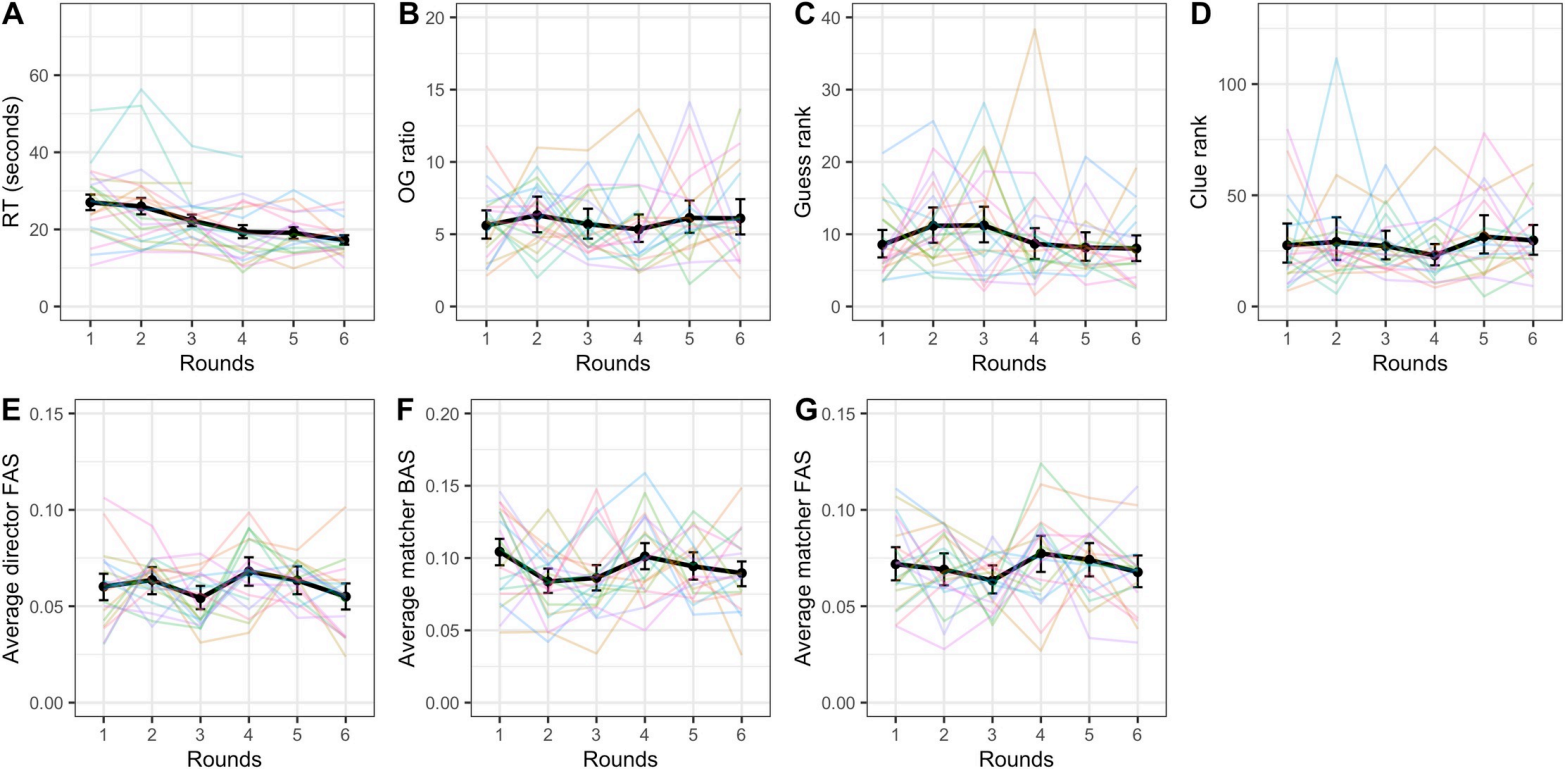
Reaction time (A), ratio between optimal clue and the clue given (B), guess rank (C), clue rank (D), director forward association strength (E), matcher backward association strength (F), and matcher forward association strength (G) across rounds. The black lines indicate average across dyads with error bars showing bootstrapped 95% confidence intervals. Colored lines represent individual dyads. Note the different scales.

None of the other dependent variables (director and matcher forward association strength, matcher backward association strength, clue rank, guess rank, and ratio between the optimal clue and the clue given) showed significant improvement across rounds (see Table 3 for the random effects structure and statistics). Reaction time did however change over rounds (see Fig 6 for visual representations of all secondary dependent variables). A linear mixed-effects model with by-dyad random slope by round and (uncorrelated) random intercept and by-target random intercept indicated that round was a significant predictor of reaction time with participants getting 1.45 seconds faster on the average trial time per round (*β* = -1.45; SE = 0.25; p < .001). Additionally, a linear mixed-effects model of accessibility predicting reaction time (random effects: by-dyad and by-target word random slopes over rounds and uncorrelated random intercept for dyad) showed that the 3^rd^ and 4^th^ quantiles were significantly faster than the baseline 1^st^ quantile (1^st^ vs 2^nd^: β = -0.03, SE = 1.00, p = 0.976; 1^st^ vs 3^rd^: β = -4.44, SE = 1.02, p < .001; 1^st^ vs 4^th^: β = -6.35, SE = 1.01, p < .001).

**Table 3:** Statistical models summary table (Experiment 1).

Dependent variable	Random effects structure	β	SE	p
Director forward association strength	Random slope for round by director and by target word, by-target word random intercept	-0.0005	0.0007	.505
Matcher forward association strength	Random slope for round by matcher and by target word	0.0002	0.001	.893
Matcher backward association strength	Random slope for round by matcher and by target word, random intercept by target word (correlated)	-0.001	0.001	.333
Clue rank	By-director random slope for round, random intercept by target	0.43	1.02	.673
Guess rank	Random slope by matcher and by target over rounds	-0.38	0.28	.176
Ratio between optimal clue and the clue given	Random slope for round by director and by target word, by-target word random intercept	0.12	0.15	.412

SE = Standard Error.

### 2.4 Discussion

Over the course of the word guessing game, matchers were able to guess around a third of the target words. Although quite low in absolute terms, given how open-ended the task was, this actually represents an impressive level of performance. We quantify the baseline success rate if participants only relied on their own perspective to solve the task, i.e., if the director provided the clue word with the forward association strength given the target word and the matcher likewise made the guess with the highest forward association strength given that clue (e.g., the director sees the target word ‘bird’ and provides the first word that comes to mind as a clue word, namely ‘fly’; the matcher in turn sees the clue word ‘fly’ and guesses the first word that comes to mind, i.e., ‘plane’; this results in an unsuccessful trial). In Experiment 1, the baseline success rate would be 8.47%, which is less than a third of our observed success rate. This strongly suggests that participants were able to engage in perspective-taking in the task. Accessibility also predicted success with the most accessible target words (targets with good potential clue words) being significantly easier for directors to convey and matchers to guess, indicating that directors were sensitive to the existence of good clue words from an allocentric perspective, again suggesting perspective-taking in the task. However, there was also evidence that perspective-taking was difficult: Symmetry predicted success, with top 1 symmetric items being significantly easier to communicate successfully than top 3 symmetric or asymmetric targets, suggesting that directors found the task easiest on trials where perspective-taking was not required to succeed.

It is worth noting that we assume that the population-level word association norms taken from the SWOW capture or reflect the associations of the individual participants in our experiment; it could instead be argued that perspective-taking as we measure it (via backward and forward association strength) actually reflects the ‘typicality’ of both participants’ word associations instead of their modelling the potentially idiosyncratic associations of their partner. However, 1) both accessibility and symmetry were derived from population-level norms and were significant predictors of success for our dyads, showing that the population-level association norms were indeed relevant, and 2) while our measures of perspective-taking (backward and forward association strength) may be reliant on population norms, the success measure is not–thus if participants were attuning to each other’s specific perspectives and not the population norms, we would expect to see improvement in success with no concomitant improvement in backward association strength–but we do not.

Even though participants’ baseline success rate is impressive, contrary to our hypothesis we found that participants’ perspective-taking did not improve over time, either on a performance-based measure of success, or on a more fine-grained measure of perspective-taking (e.g., director backward association strength). This is somewhat surprising, given that participants in Sulik and Lupyan’s study [35] improved over time with the same level of feedback as in the present experiment, and might suggest that there are limits on participants’ ability to adjust perspective-taking during interaction. However, there may be factors in the design of our experiment that limited participants’ ability to show improvement in perspective-taking over time. In particular, we wondered whether the search for potential clue words (highlighted by Sulik & Lupyan [29], as a factor) was too demanding and prevented improvement over time. Alternatively, it may be that our task was too opaque, and participants were unable to generate alternative strategies which would allow them to improve over time, in particular strategies that involved consistent perspective-taking rather than other lower-level heuristics. To test whether these factors limited performance, in Experiment 2 we test whether performance could improve with richer feedback and more accessible targets.

## 3. Experiment 2

**Fig 7:**
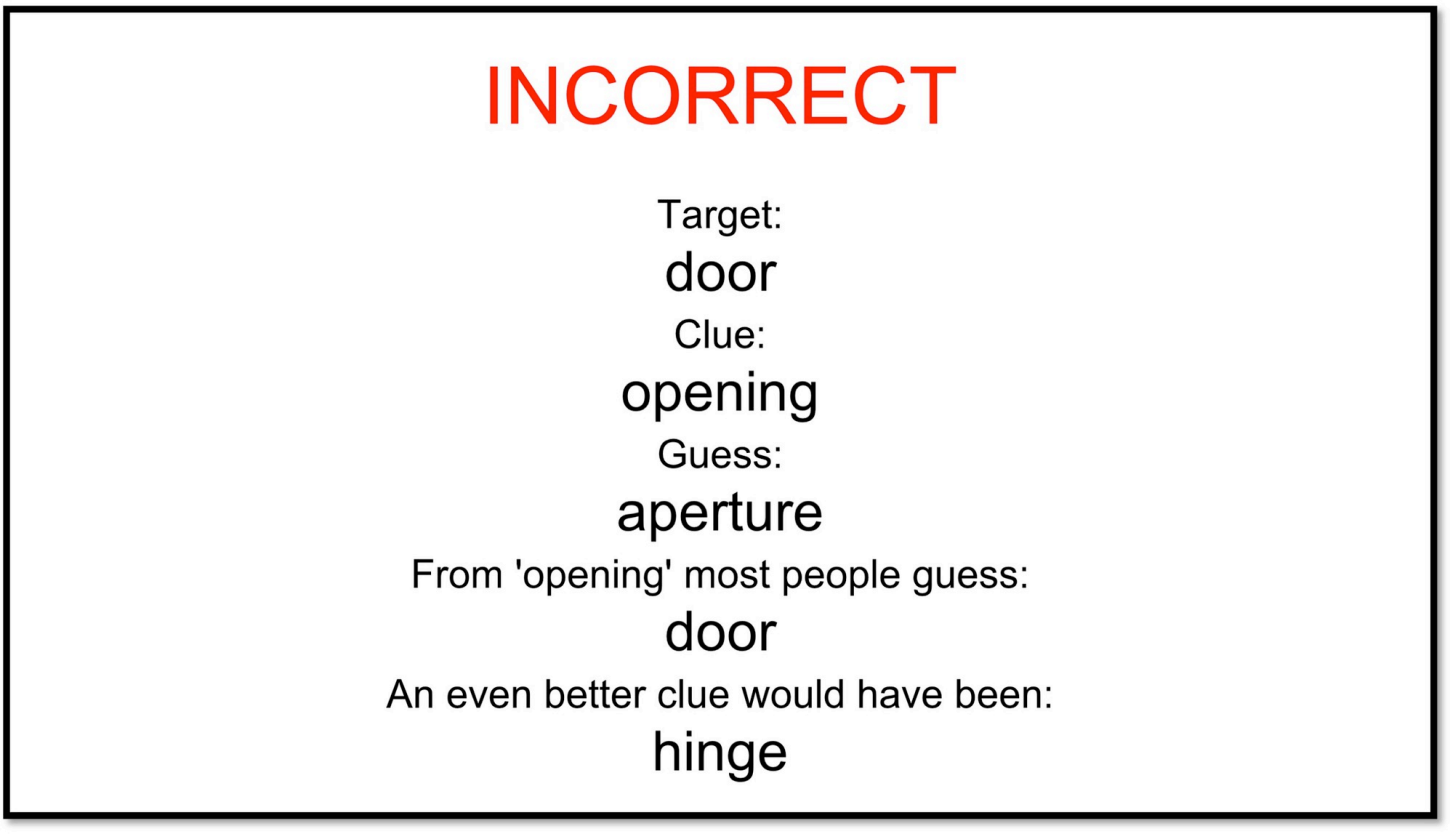
An example of the feedback screen after an incorrect trial in Experiment 2.

If genuine perspective-taking is effortful, we would expect it to be more accessible to participants when the task is easier and participants are explicitly instructed on what makes a good clue word, focusing on their partner’s perspective. We therefore expanded the trial-by-trial feedback in Experiment 2 so that participants were also told after unsuccessful trials what the optimal clue word would have been, and what guess people usually provide in response to the clue the director actually gave (see Fig 7 for an example of the feedback screen in Experiment 2). Given previous literature showing that explicitly instructing participants to take perspective substantially improves performance [46], the more extensive feedback should also be helpful to our participants. This is similarly supported by beneficial effects of constraining the search space (e.g., [29]), effectively showing participants what good clues are like. We created a new set of target words which all had good potential clue words (optimal backward association strength ranging from 0.23 to 0.38 compared with from 0.01 to 0.33 in Experiment 1) to test whether the lack of improvement in Experiment 1 was due to the difficult task of searching for and simulating responses to clue words. Furthermore, we decided to only use asymmetric items in Experiment 2 to make it clearer what would be a good strategy: the mixture of symmetric and asymmetric target words in Experiment 1 could have added to confusion about the optimal strategy; on symmetric trials, participants succeeded using the most salient clue word from their own perspective, while on asymmetric trials, the better strategy was for the director to suppress her own perspective and search for the clue word that would make the target word most salient from the matcher’s perspective. In sum, the design of Experiment 2 should be maximally helpful in eliciting perspective-taking, and allow participants to develop their perspective-taking over the course of the experiment thanks to richer feedback and a set of targets which consistently reward perspective-taking. Apart from the changes described here, everything else about the experiment procedure (i.e., the inclusion criteria, timings, compensation) was the same as in Experiment 1.

### 3.1 Method

#### Participants

For Experiment 2, we again recruited 40 participants from the student population at the University of Edinburgh, playing as 20 dyads (12 male and 28 female, mean age = 23.33, range = 19–52). As in Experiment 1, the participants in pairs did not know each other before participating in the experiment, and dyad allocation was based on which timeslot participants signed up for. There were 14 same-gender dyads and 6 different-gender dyads, and the difference in age within a dyad ranged from 0 to 30 years (median = 3 years).

#### Materials

We selected 120 new target words from the most accessible words (the words with the highest conditional probabilities given potential clue words) in the SWOW dataset in an attempt to make the task less challenging. None of the target words had top 1 symmetric associates but they could have top 3 symmetric associates.

#### Procedure

Aside from the on-screen instructions which were the same as in Experiment 1, participants were given examples of trials demonstrating successful and unsuccessful perspective-taking orally by the experimenter (see Experiment 1 procedure for more detail). Experiment 2 also featured richer feedback after each trial than provided in Experiment 1: as well as being informed of success/failure, the target, and the matcher’s guess, after unsuccessful trials participants were additionally told what the optimal clue word would have been (based on backwards association strength) and the top associate in response to the clue the director actually gave (see Fig 7 for an example of the changed feedback screen in Experiment 2). The 120 optimal clue words were calculated from the SWOW dataset prior to the experiment’s start, and the ‘most people guess’ words were calculated as the experiment progressed, likewise from the SWOW dataset. If the clue did not appear in the SWOW dataset, both director and matcher saw ‘UNKNOWN’ instead of the top associate in ‘From [clue] most people guess: [top associate]’. They were instructed that ‘UNKNOWN’ meant that it was not possible to look up the information based on their clue word.

### 3.2 Results

The dependent and independent variables were identical to Experiment 1, excluding the symmetry variable which was unnecessary as all target words in Experiment 2 had asymmetric associates. In Experiment 2 where all the target words were selected to have good potential clue words, the maximal backward association strength had an overall mean of 0.30 and ranged from 0.23 to 0.38. The 1^st^ quartile was 0.28 and the 3^rd^ quartile was 0.31.

#### Data cleaning and preparation

One dyad only played three rounds, and two dyads only played five rounds. Their trials were still included in the analyses. Three trials in total were excluded from the analyses due to technical errors while recording responses. As in Experiment 1, we were not able to look up information for all the clue and guess words. This was the case for 325 of the trials for director forward association strength (14.21%), 425 of the trials for matcher forward association strength (18.58%), 380 of the trials for director backward association strength, clue rank, and ratio between optimal clue and the clue given (16.62%), and 408 of the trials for matcher backward association strength and guess rank (17.84% of the trials). Again, there appeared to be no clear pattern as to whether these proportions of associations that could not be looked up changed over time. As in Experiment 1, there were only a few cases where participants used disallowed words, those occurred early in the experiment, and in those cases, the experimenter interrupted the session, repeated the instructions, and restarted the experiment from the beginning.

#### Descriptive statistics

**Fig 8:**
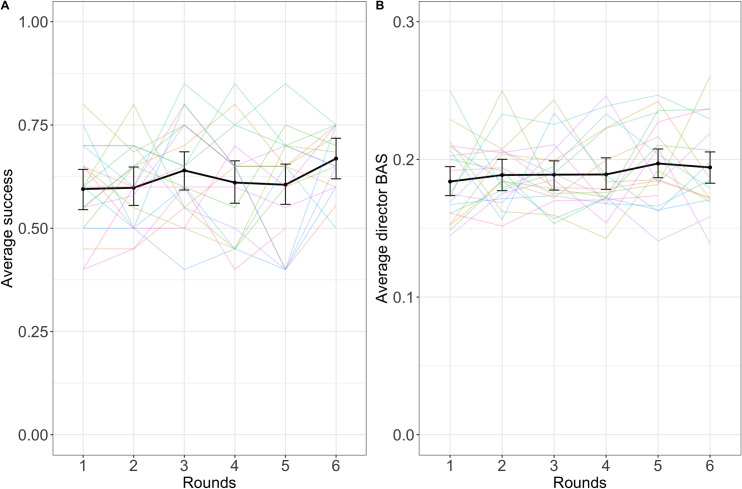
Success (A) and director backward association strength (B) over rounds. The black lines indicate averages across dyads with error bars showing bootstrapped 95% confidence intervals. Colored lines represent individual dyads. Note the different scales in A and B.

See Fig 8A for average success over rounds and Fig 8B for average director backward association strength over rounds. See Table 4 for the numerical descriptive statistics of all the dependent variables across rounds.

**Table 4:** Means and standard deviations of success, RT (reaction time in seconds), director backward association strength (BAS), director forward association strength (FAS), matcher backward association strength (BAS), matcher forward association strength (FAS), clue rank, guess rank, and optimal:given (OG) ratio.

Round	Success	RT	Director BAS	Director FAS	Matcher BAS	Matcher FAS	Clue rank	Guess rank	OG ratio
1	0.60 (*0*.*49*)	28.10 (*21*.*09*)	0.184 (*0*.*10*)	0.06 (*0*.*05*)	0.17 (*0*.*11*)	0.06 (*0*.*06*)	15.58 (*34*.*78*)	4.63 (*12*.*17*)	6.15 (*15*.*42*)
2	0.60 (*0*.*49*)	26.52 (*21*.*27*)	0.189 (*0*.*10*)	0.06 (*0*.*05*)	0.17 (*0*.*11*)	0.06 (*0*.*06*)	20.41 (*113*.*35*)	5.95 (*15*.*43*)	5.23 (*12*.*98*)
3	0.64 (*0*.*48*)	24.94 (*18*.*71*)	0.189 (*0*.*10*)	0.06 (*0*.*05*)	0.18 (*0*.*10*)	0.06 (*0*.*06*)	12.82 (*23*.*56*)	4.50 (*11*.*82*)	4.18 (*10*.*59*)
4	0.61 (*0*.*49*)	23.68 (*17*.*27*)	0.189 (*0*.*10*)	0.05 (*0*.*05*)	0.18 (*0*.*11*)	0.06 (*0*.*06*)	19.48 (*109*.*84*)	4.68 (*11*.*92*)	4.90 (*12*.*42*)
5	0.61 (*0*.*49*)	23.41 (*15*.*48*)	0.197 (*0*.*10*)	0.05 (*0*.*05*)	0.18 (*0*.*10*)	0.06 (*0*.*05*)	11.83 (*31*.*62*)	4.03 (*11*.*00*)	4.30 (*12*.*12*)
6	0.67 (*0*.*47*)	21.02 (*13*.*62*)	0.194 (*0*.*10*)	0.06 (*0*.*05*)	0.18 (*0*.*11*)	0.06 (*0*.*06*)	9.54 (*18*.*38*)	3.72 (*8*.*77*)	3.48 (*9*.*37*)

#### Success

A binomial mixed-effects regression model with random slopes by round for each dyad and random slopes and intercepts by round for each target word indicated a marginally significant positive effect of round on success (β = 0.07, SE = 0.04, p = .081).

**Fig 9:**
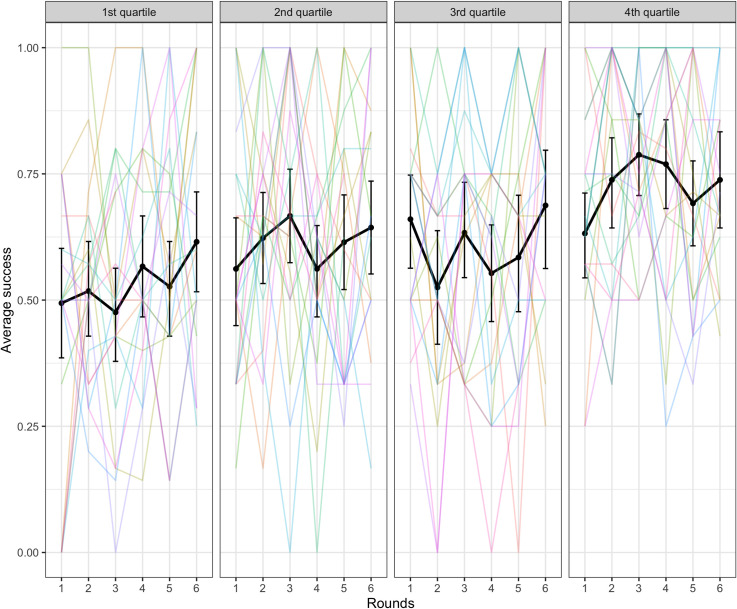
Success in the word guessing game by accessibility of the target word over rounds. The black lines indicate average across dyads with error bars showing bootstrapped 95% confidence intervals. Colored lines represent individual dyads.

We ran a generalized linear mixed-effects model of accessibility predicting success with random slope over rounds by dyad, random slope and intercept over rounds by target word, and (uncorrelated) random intercept by dyad. This model indicated that accessibility was again a significant predictor of success with the 4^th^ quartile being significantly easier to communicate than the baseline 1^st^ quartile (1^st^ vs 2^nd^: β = 0.40, SE = 0.26, p = .121; 1^st^ vs 3^rd^: β = 0.34, SE = 0.27, p = .216; 1^st^ vs 4^th^: β = 0.99, SE = 0.26, p < .001). See Fig 9. The differences between quartiles were smaller in Experiment 2 compared with Experiment 1 which is not surprising given that all the target words were selected to be accessible in Experiment 2.

#### Director backward association strength

A linear mixed-effects regression model with random slopes by round for each participant, uncorrelated random intercept for each participant, and random slopes and intercepts by round for each target word indicated a small but significant positive effect of round on director backward association strength (β = 0.003, SE = 0.002, p = .043).

#### Further measures of improvement

**Fig 10:**
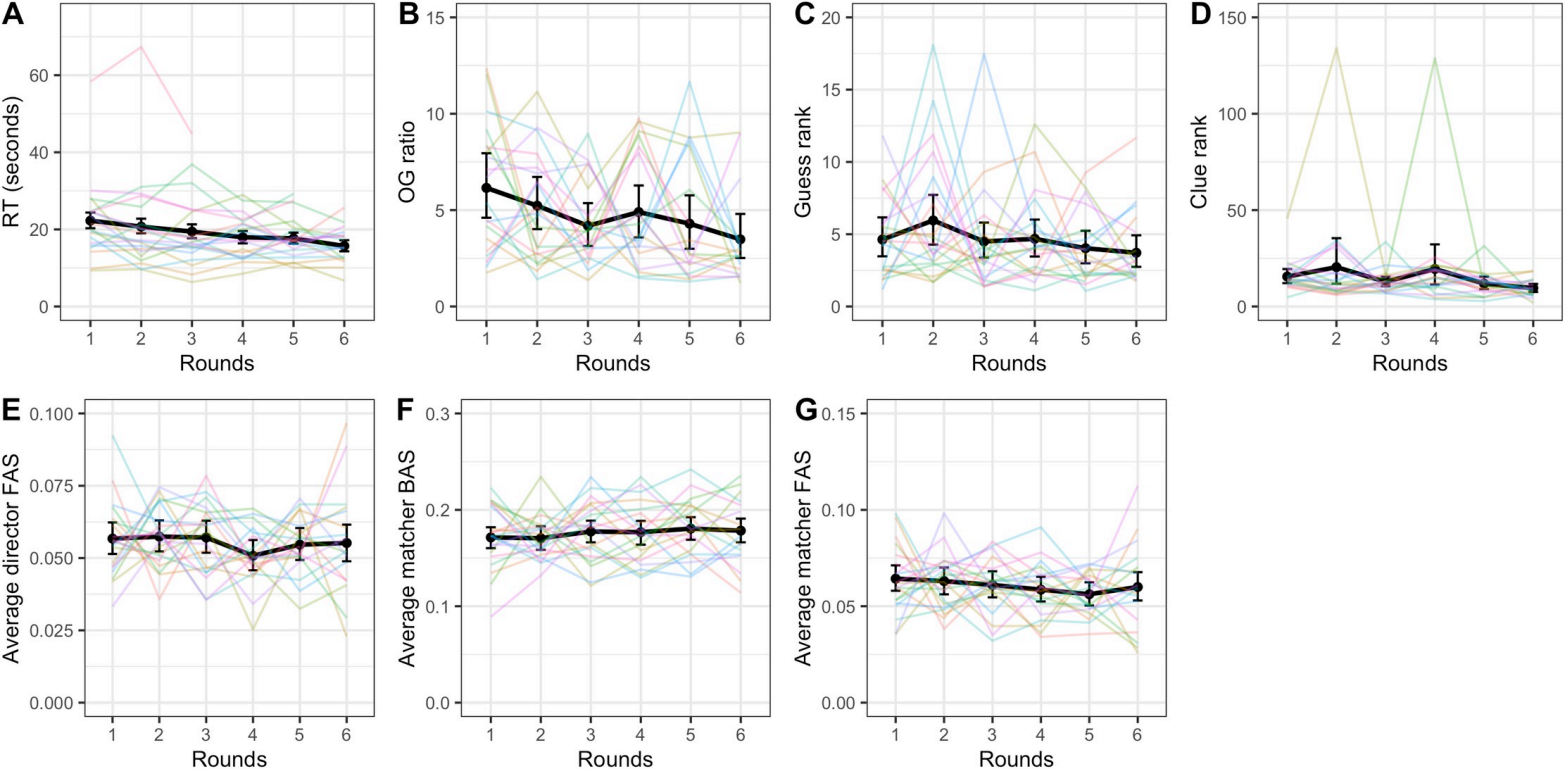
Reaction time (A), ratio between optimal clue and the clue given (B), guess rank (C), clue rank (D), director forward association strength (E), matcher backward association strength (F), and matcher forward association strength (G) across rounds. The black lines indicate average across dyads with error bars showing bootstrapped 95% confidence intervals. Colored lines represent individual dyads. Note the different scales.

The secondary dependent variables matcher forward association strength and ratio between optimal clue and the clue given both appeared to change significantly over rounds (see Fig 10 for visual representations of all the dependent variables and Table 5 for summary statistics and random effects structures). A model of matcher forward association strength predicted by round suggested that this measure decreased over rounds, again indicating less reliance on egocentric salience in matchers (β = -0.002; SE = 0.0008; p = .014). Director forward association strength (β = -0.001; SE = 0.0006, p = .063) and guess rank (β = -0.30; SE = 0.17; p = .069) both showed marginally significant reduction, indicating less reliance on egocentric salience in generating clues and a trajectory towards better guesses, respectively. Matcher backward association strength and clue rank did not appear to improve over rounds (see Table 5). As in Experiment 1, accessibility was also a significant predictor of reaction time, with the 4^th^ quantile being significantly faster than the baseline 1^st^ quantile (2^nd^: β = -1.23, SE = 1.07, p = .255; 3^rd^: β = -0.51, SE = 1.12, p = .650; 4^th^: β = -2.62, SE = 1.08, p = .016). This model included random slopes over round by target word and by dyad as well as uncorrelated random intercept for dyad.

**Table 5:** Statistical models summary table (Experiment 2).

Dependent variable	Random effects structure	*β*	SE	p
Director forward association strength	Random slope for round by director, by-target word and by-director random intercept	-0.001	0.0006	.063^+^
Matcher forward association strength	Random intercept by matcher and by target word	-0.002	0.0008	.014*
Matcher backward association strength	Random slope for round by matcher and by target word, random intercept by target word (correlated)	0.003	0.002	.116
Clue rank	Random intercepts for director and for target word	-1.42	0.93	.126
Guess rank	Random intercepts for director and for target word	-0.30	0.17	.069^+^
Ratio between optimal clue and the clue given	Random intercepts for director and for target word	-0.44	0.16	.007*

SE = Standard Error. ‘*’ indicates that round was a significant predictor of the dependent variable in question, ‘+’ indicates that round was a marginally significant (p = < .1) predictor of the dependent variable in question.

#### Zipf value

**Fig 11:**
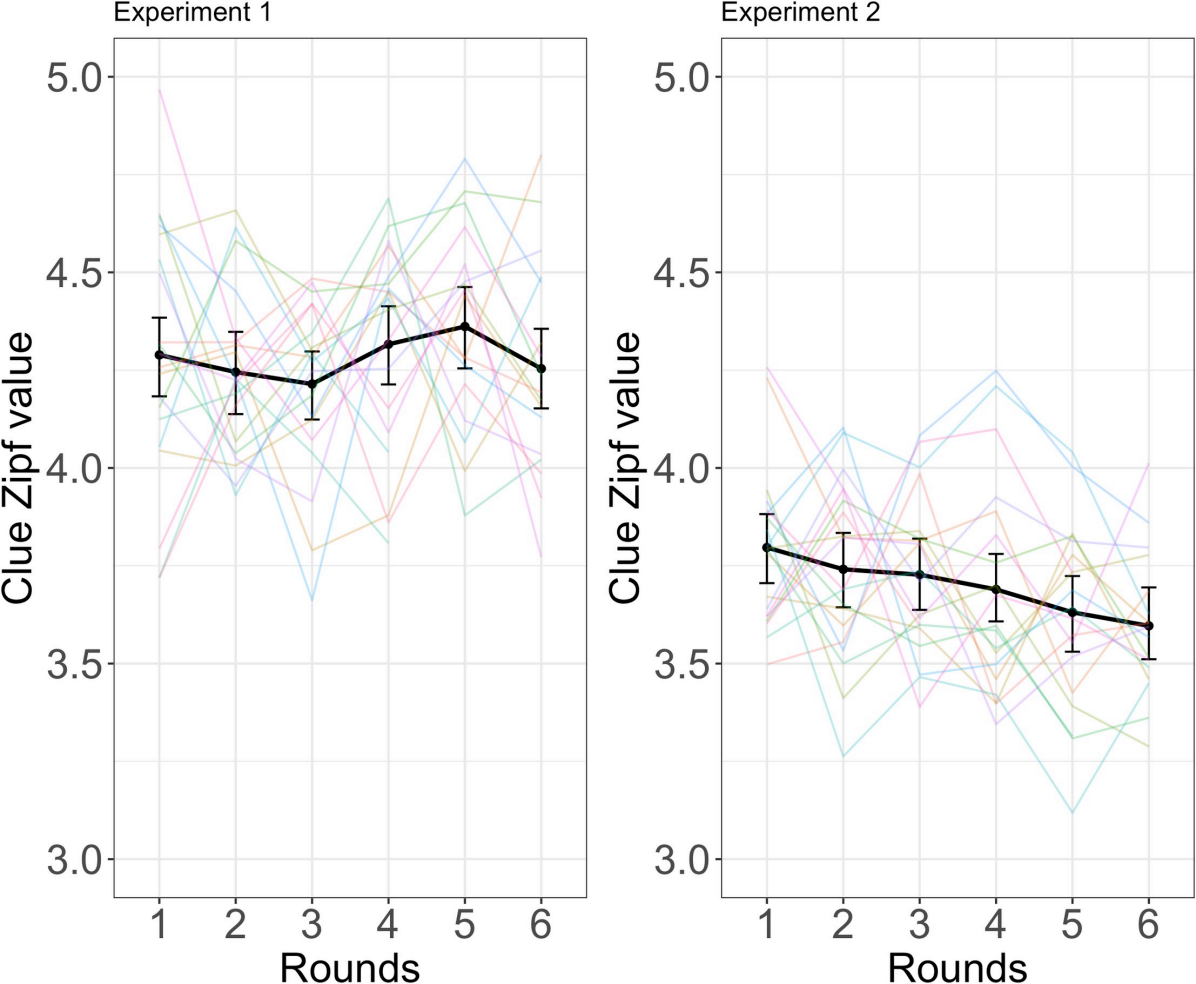
An illustration of Zipf value (clue word frequency) over rounds in Experiment 1 (left) and Experiment 2 (right).

During the analysis of the Experiment 2 data, we noticed that the suggested clues given in the feedback after incorrect trials were often quite rare words. As an exploratory analysis, we therefore tested the development of clue word frequencies over the course of the game, operationalized by Zipf value [47]. The higher the Zipf value, the more common the word: A value of 1 corresponds to words with frequencies of 1 per 100 million words, a value of 2 corresponds to frequencies of 1 per 10 million, a value of 3 to frequencies of 1 per million words, etc. A linear mixed-effects model of Zipf value predicted by round indicated directors produced clue words with lower Zipf value–i.e., lower frequency–with every round (β = -0.04; SE = 0.01; p < .001). This model included random slope over rounds by director and uncorrelated random intercepts for director and target word. When we ran the same type of model on the Experiment 1 data, round did not predict Zipf value (β = 0.002; SE = 0.01; p = .891). This model included random intercepts for director and target word. See Fig 11 below for a visualization of Zipf value over rounds in the two experiments.

### 3.3 Discussion

Experiment 2 was designed to be easier than Experiment 1: we provided richer feedback, selected targets for which good clue words existed, and selected targets for which a consistent strategy was appropriate (although since we used asymmetric targets throughout this was the harder perspective-taking strategy). Participants in Experiment 2 showed higher levels of success than in Experiment 1 (succeeding on average in two thirds of all trials rather than one third). As in Experiment 1, we again quantified the baseline success rate if participants only relied on their own perspective to solve the task, i.e., if director and matcher both selected the word with the highest forward association strength from their perspective. In Experiment 2, the baseline success rate would be 10.27%, approximately 1/6^th^ of the observed success rates. Our participants were therefore clearly using some perspective-taking. As in Experiment 1, accessibility was a significant predictor of success, once again indicating that participants were sensitive to the existence of good and bad clue words.

In contrast to Experiment 1, there was some indication that participants were able to slightly improve their performance over the course of the game: success, director forward association strength, and guess rank all showed marginally significant improvement while director backward association strength, matcher forward association strength, and ratio between optimal clue and clue given all improved significantly. This might suggest that participants fine-tuned their perspective-taking in this task, either adopting a perspective-taking strategy more often or improving their ability to estimate their partner’s perspective. However, an exploratory analysis using word frequency (Zipf value) of the clue words suggested that participants changed over time to use rarer clue words, suggesting the alternative explanation that directors simply realized that picking less common clue words would improve success. Importantly, we cannot definitively exclude either a perspective-taking explanation or a frequency-based heuristics explanation for the slight improvement found in Experiment 2.

Identifying what features of Experiment 2 made this improvement over time possible is complicated by the fact that we made two changes to the design of Experiment 1: we used only asymmetric targets with a good clue available, and we provided richer feedback. Plausibly either of these could have allowed participants to identify and exploit a consistent perspective-taking strategy, or indeed a more superficial strategy based on using low-frequency clues. We therefore conducted a third experiment where we used the same set of target words as in Experiment 1 (i.e., involving a wider range of difficulties and a mix of symmetric and asymmetric targets) with the extended feedback of Experiment 2, to attempt to identify which of these features was responsible.

## 4. Experiment 3

### 4.1 Method

#### Participants

Due to the 2020 coronavirus pandemic, we were unable to run a lab-based experiment. For Experiment 3, we therefore recruited 90 participants playing as dyads through the online behavioral experiments platform Prolific, with the inclusion criterion that they had English as their self-reported first language (as given by the Prolific screening tools). Out of the 90 participants who started the experiment on Prolific, 40 participants organized into 20 dyads completed more than two rounds without network errors (e.g., caused by one member of a dyad dropping out) and only data from these 40 were included in subsequent analyses (20 male and 20 female, mean age = 36.20, range = 21–62). As in Experiments 1 and 2, the participants in pairs did not know eachother beforehand, and allocation to dyads was based on the order in which participants entered the experiment virtual waiting room (as soon as a participant arrived in the virtual waiting room they were paired with the participant waiting, clearing the waiting room, or waited for a second participant to arrive). Participants were paid £10 upon completing the experiment or compensation if their partner dropped out or there was a technical error (£2 if they had played for less than 10 minutes and £5 if they had played for more than 10 minutes). There were 14 same-gender dyads and 6 different-gender dyads, and the difference in age within a dyad ranged from 1 to 37 years (median = 13 years).

#### Materials

The stimulus materials were identical to the ones used in Experiment 1, featuring target words that were quite different in terms of accessibility (the existence of good clue words) and which differed in how much they required perspective-taking (half were symmetric, half were asymmetric). We built the experiment using JavaScript (for the participant-side part of the experiment running in a web browser) and Python (for a server coordinating communication across participants paired to form a dyad).

#### Procedure

Participants received the richer feedback provided in Experiment 2, including examples of good clues and likely guesses based on the clue provided (see Fig 7 for an example). The instructions were the same as in Experiments 1 and 2.

### 4.2 Results

#### Data cleaning and preparation

Of the 40 participants playing as 20 dyads, only one dyad did not complete all six rounds but instead completed four. Data from all these 40 participants were included in subsequent analyses; trials where they had failed to follow instructions (for example by providing more than one clue word) were excluded from analysis, resulting in the exclusion of 97 trials in total (4.32%). As in Experiments 1 and 2, we were not able to look up information for all the clue and guess words. This was the case for 451 of the trials for director forward association strength (20.08%), 607 of the trials for matcher forward association strength (27.03%), 506 of the trials for director backward association strength (22.53%), 492 of the trials for matcher backward association strength (21.91% of the trials), and 284 of the trials for Zipf value (12.64%). There was no clear pattern as to whether these proportions of associations that could not be looked up changed over time.

#### Descriptive statistics

**Fig 12:**
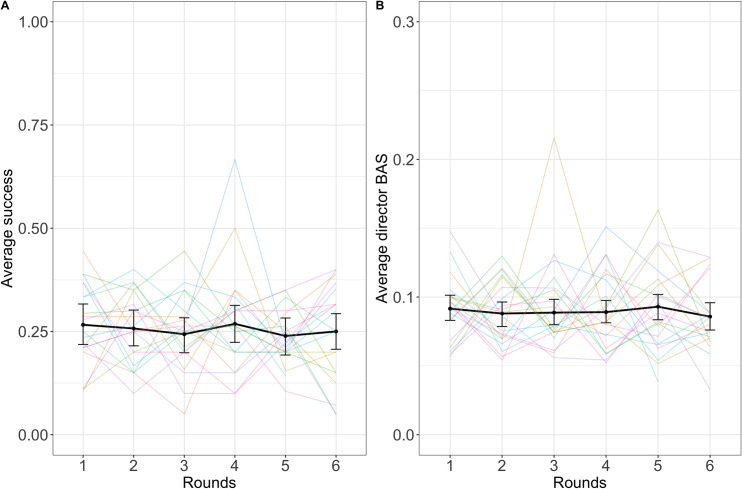
Success (A) and director backward association strength (B) over rounds. The black lines indicate averages across dyads with error bars showing bootstrapped 95% confidence intervals. Colored lines represent individual dyads. Note the different scales in A and B.

See Fig 12A for average success over rounds and Fig 12B for average director backward association strength over rounds. See Table 6 for means and standard deviations of all the dependent variables and Fig 14 for visualizations of all the dependent variables.

**Table 6:** Means and standard deviations of success, RT (reaction time in seconds), director backward association strength (BAS), director forward association strength (FAS), matcher backward association strength (BAS), matcher forward association strength (FAS), clue rank, guess rank, optimal:given (OG) ratio, and Zipf value across rounds.

Round	Success	RT	Director BAS	Director FAS	Matcher BAS	Matcher FAS	Clue rank	Guess rank	OG ratio	Zipf value
1	0.27 (*0*.*45*)	29.78 (14.91)	0.09 (*0*.*08*)	0.06 (*0*.*06*)	0.1 (*0*.*08*)	0.07 (*0*.*07*)	35.33 (129.51)	6.18 (12.6)	6.15 (11.27)	4.38 (0.87)
2	0.27 (*0*.*45*)	30.71 (15.19)	0.09 (*0*.*08*)	0.06 (*0*.*06*)	0.09 (*0*.*08*)	0.07 (*0*.*07*)	25.71 (61.56)	9.51 (18.56)	5.79 (9.98)	4.3 (0.92)
3	0.27 (*0*.*44*)	28.8 (13.05)	0.09 (*0*.*08*)	0.06 (*0*.*07*)	0.09 (*0*.*08*)	0.07 (*0*.*08*)	30.25 (62.4)	9.67 (20.62)	5.98 (9.02)	4.3 (0.89)
4	0.28 (*0*.*45*)	27.59 (13.89)	0.09 (*0*.*08*)	0.06 (*0*.*06*)	0.1 (*0*.*08*)	0.07 (*0*.*07*)	29.44 (77.35)	8.78 (19.3)	5.64 (9.07)	4.39 (0.88)
5	0.24 (*0*.*43*)	27.72 (16.29)	0.09 (*0*.*08*)	0.07 (*0*.*07*)	0.1 (*0*.*08*)	0.08 (*0*.*07*)	31.82 (75.6)	9.58 (20.27)	5.94 (9.3)	4.4 (0.95)
6	0.25 (*0*.*43*)	26.49 (13.91)	0.09 (*0*.*08*)	0.06 (*0*.*06*)	0.08 (*0*.*07*)	0.07 (*0*.*08*)	38.52 (98.1)	12.19 (22.81)	7.05 (11.13)	4.38 (0.92)

#### Success

We analyzed success predicted by round number with a binomial mixed-effects regression model and found no evidence of an effect of round on success (β = -0.002, SE = 0.03, p = .955). This model included random intercepts for dyad and target word. This matches the similar finding from Experiment 1.

**Fig 13:**
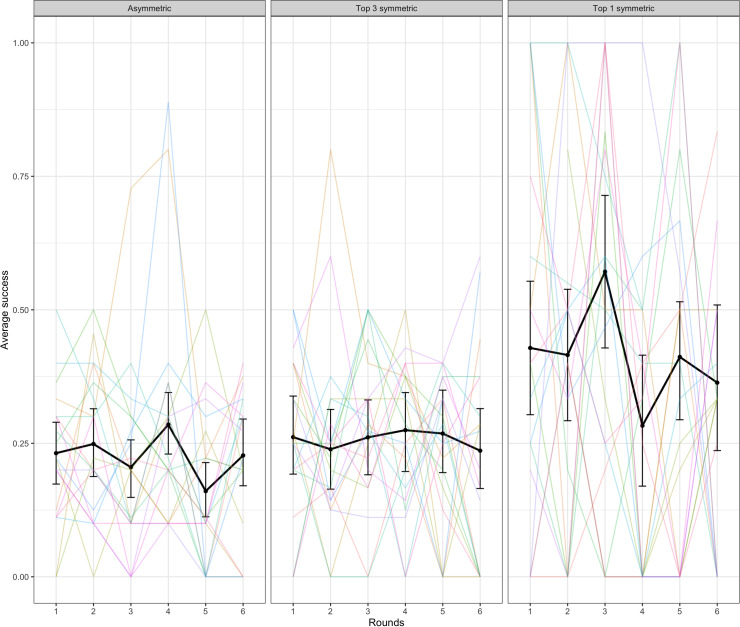
Success in the word guessing game by symmetry of the target word. The black lines indicate average across dyads with error bars showing bootstrapped 95% confidence intervals. Colored lines represent individual dyads.

A binomial mixed-effects regression model with success predicted by symmetry indicated that top 1 symmetric target words were significantly easier to communicate than asymmetric targets words (β = 1.00, SE = 0.32, p = .002). Top 3 symmetric targets were not significantly easier compared with asymmetric targets (p = .404). See Fig 13. This matches the similar finding from Experiment 1.

**Fig 14:**
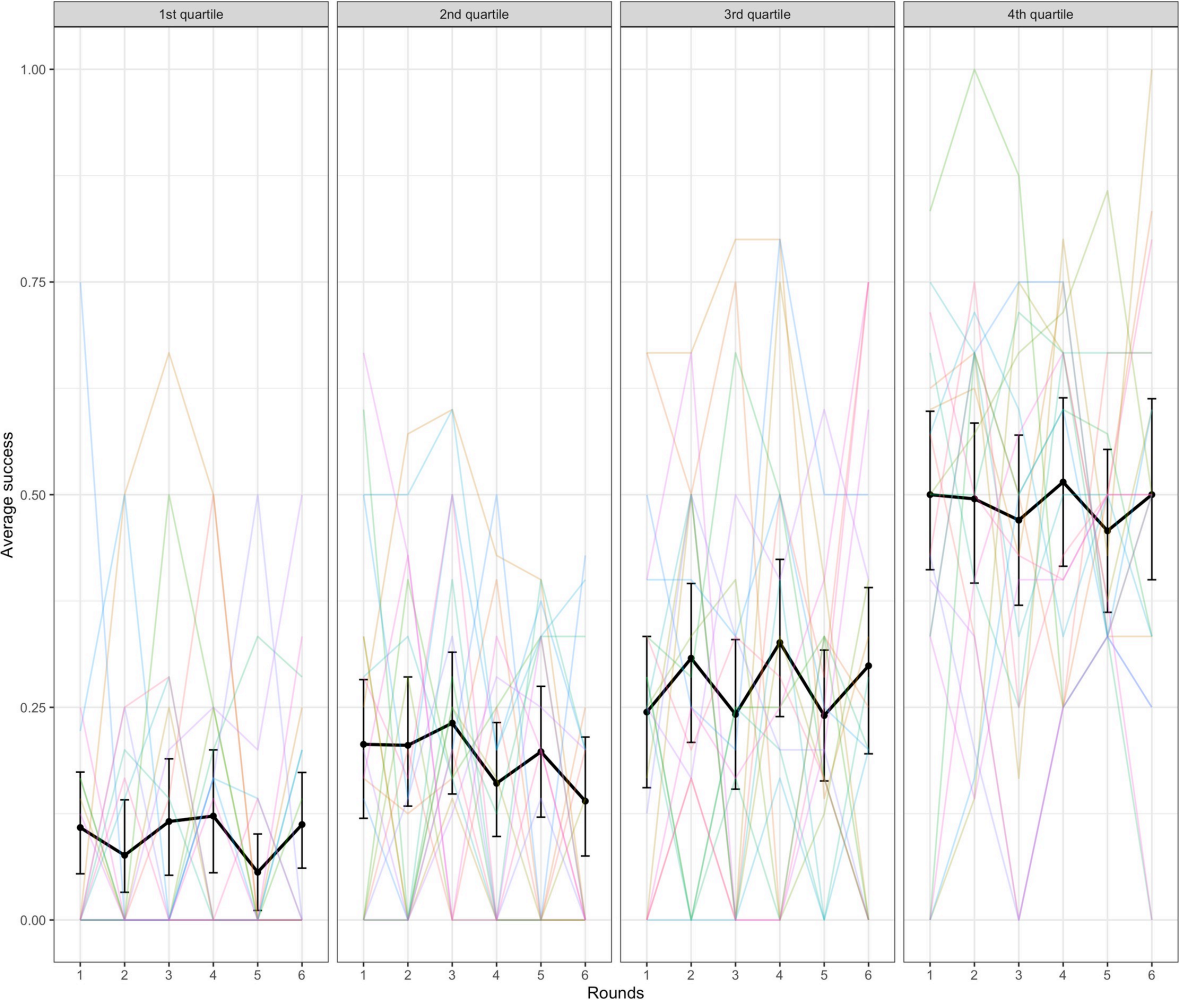
Success in the word guessing game by accessibility of the target word. The black lines indicate average across dyads with error bars showing bootstrapped 95% confidence intervals. Colored lines represent individual dyads.

Accessibility was also a significant predictor of success with the 2^nd^, 3^rd^ and 4^th^ quartiles being easier to communicate than the baseline 1^st^ quartile (1^st^ vs 2^nd^: β = 0.85, SE = 0.25, p < .001; 1^st^ vs 3^rd^: β = 1.41, SE = 0.25, p < .001; 1^st^ vs 4^th^: β = 2.43, SE = 0.25, p < .001). See Fig 14. This matches the similar finding from Experiment 1.

#### Director backward association strength

A linear mixed-effects regression model indicated no effect of round on director backward association strength (β = 0.0002, SE = 0.0008, p = .812). This model included random intercepts for director and target word. This matches the similar finding from Experiment 1.

#### Further measures of improvement

**Fig 15:**
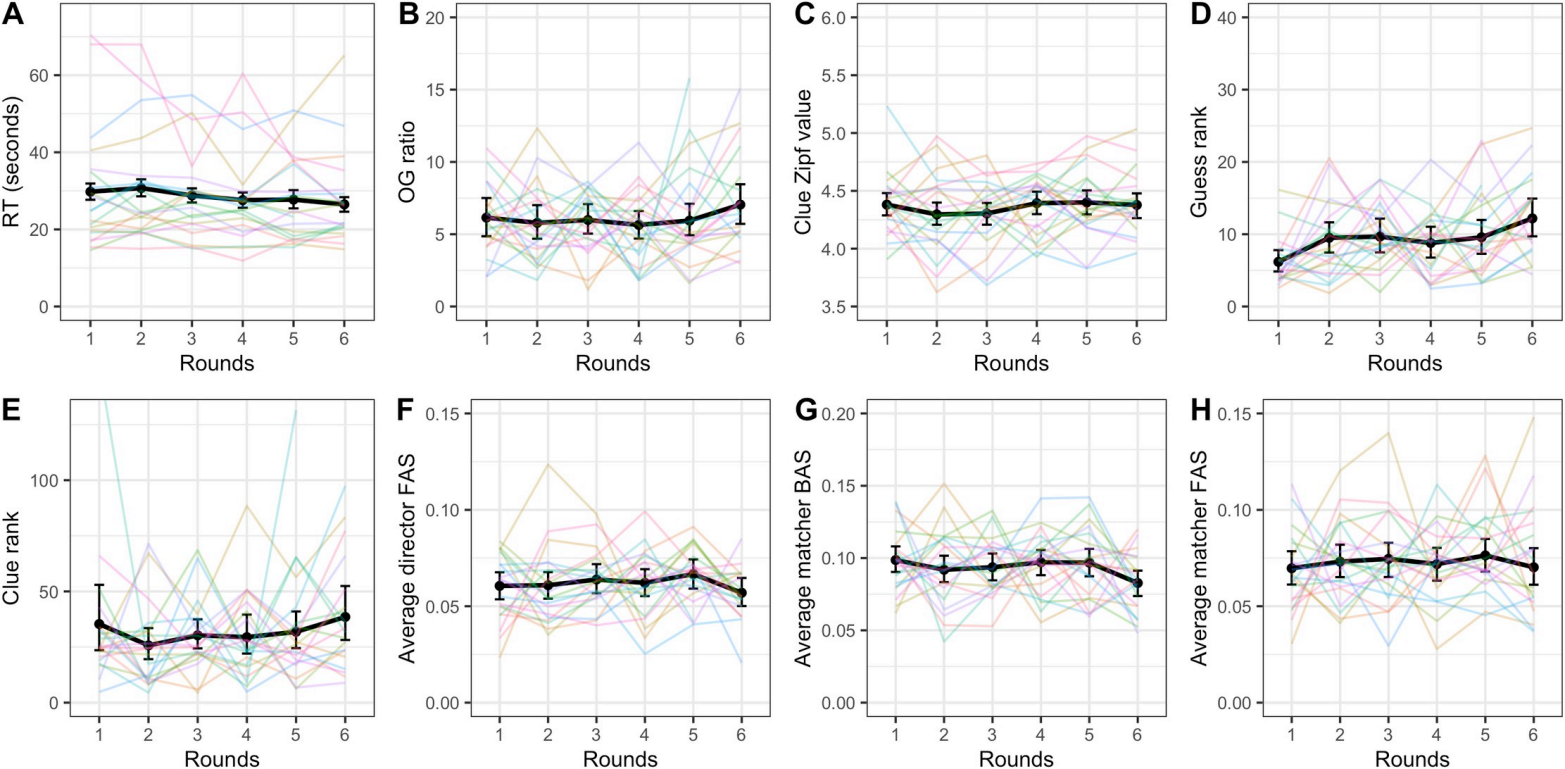
Reaction time (A), ratio between optimal clue and the clue given (B), clue Zipf value (C), guess rank (D), clue rank (E), director forward association strength (F), matcher backward association strength (G), and matcher forward association strength (H) across rounds. The black lines indicate average across dyads with error bars showing bootstrapped 95% confidence intervals. Colored lines represent individual dyads. Note the different scales.

None of the other dependent variables (Zipf value, director and matcher forward association strength, matcher backward association strength, clue rank, guess rank, ratio between optimal clue and the clue given) showed significant improvement across rounds. See Table 7 for a summary of the models run to test these relationships and Fig 15 for visualizations. Guess rank significantly increased over rounds but this means guesses became worse, not that they became better.

**Table 7:** Statistical models summary table (Experiment 3).

Dependent variable	Random effects structure	*β*	SE	p
Director forward association strength	Random intercept for director and target word	-0.00006	0.0007	.932
Matcher forward association strength	Random intercept for matcher and target word	0.0002	0.001	.825
Matcher backward association strength	Random intercept for target word	-0.001	0.001	.21
Clue rank	Random intercept for director and target word	0.80	1.23	.513
Guess rank	Random slope for matcher	0.86	0.27	.001*
Ratio between optimal clue and the clue given	Random intercept for director and target word	0.11	0.13	.426

SE = Standard Error. ‘*’ indicates that round was a significant predictor of the dependent variable in question.

### 4.3 Discussion

Despite taking place online, the results of Experiment 3 were very similar to the results of Experiment 1 which used the same 120 target words–participants succeeded on around a quarter of the trials throughout but did not improve over rounds according to any of our measures of perspective-taking. The overall success rate was thus slightly lower than in Experiment 1 (with the same target words) but more than three times higher than the baseline success rate of 8.47%. Despite the increased feedback (what would have been a better clue and what people usually guess when provided with the clue the director gave), participants in Experiment 3 did not appear to discover perspective-taking or frequency-based heuristics which allowed them to improve success as seen in Experiment 2. This is supported by the finding that clue Zipf value did not change over time in Experiment 3. Judging by the results from Experiment 3, it appears it was the higher accessibility of the target words and/or the fact that they all had asymmetric associates in Experiment 2 that caused the improvement across the game, and not the richer feedback. These possibilities are discussed in more detail below.

## 5. General discussion

The results of Experiments 1 and 3 indicated that although participants were performing well above baseline in the word guessing game, they were unable to improve their success rate and perspective-taking over time, even when provided with extensive feedback which was intended to highlight the perspective-taking aspects of the task (what people usually guess in response to the clue the director gave; what would have been a better clue). Experiment 2 provided some limited evidence that participants were able to adjust to the demands of the game under maximally helpful conditions as some of our measures of perspective-taking showed significant–but small–improvements. Here, all the target words had potentially good clue words, and participants got extensive feedback and did not have to switch strategy between symmetric and asymmetric trials because all the target words had asymmetric associates. Consistent with the previous literature on perspective-taking, symmetry was a significant predictor of success in Experiments 1 and 3, supporting the idea that it is more demanding for the director to suppress their own perspective in order to provide a useful clue. Accessibility was also a significant predictor in all three experiments, confirming that target words that had a good clue were easier to communicate than words that had weaker clues. This showed both that population-level association norms were relevant at the dyad level and that participants were sensitive to the existence of good and bad clue words throughout. It is also important to note that the participants had high success relative to an entirely egocentric baseline–getting on average around 25–33% of targets right in Experiments 1 and 3 respectively, and around 65% of the targets right in Experiment 2 –but that they appeared unable to *improve*. Below we discuss what these two findings–relatively impressive but not gradually improving performance in our paradigm–might mean in terms of the mechanisms involved in perspective-taking in the experiments as well as in real-life communication.

### 5.1 Using perspective information

We expected participants to improve by getting better at generating and evaluating clue words that strongly cued the target word. Our results contrast with those found by Sulik and Lupyan [35] where participants did improve their performance when receiving full feedback. It is difficult to ascertain what made the difference between our results and those of Sulik and Lupyan as their study is currently only accessible as a conference abstract with limited detail on the method and procedure. We cannot know why feedback helped their participants improve their perspective-taking while it did not help ours. It is possible that the difference stems from their participants interacting face to face, or perhaps it was because their participants did not switch roles and there was thus more opportunity for the director to discover a coherent perspective-taking strategy.

To interpret what our results mean for real-life communication, we should revisit the discussion of candidate cognitive subprocesses from the Introduction. We focused our attention on the ‘using perspective information’ process proposed by Apperly et al. [21] and suggested that this process may be elaborated upon based on our paradigm. Specifically, we thought that our paradigm could shed light on how people generate, evaluate, and ultimately select utterances. Given that participants in other experiments appeared able to evaluate and compare utterances for probability of success [29], it seems likely that the difficulty our participants have lies at the utterance generation stage.

### 5.2 Improving perspective-taking

Aside from the specific mechanisms involved in perspective-taking, we were also interested in whether our participants’ performance could improve with repeated interactions. In folk psychology, people have the impression that taking someone’s perspective in order to communicate more smoothly gets easier the better you know the person you are talking to. Our experimental paradigm can help answer the question of what it is that improves, if anything. In our paradigm, performance cannot improve through increasingly aligned perspectives (participants agreeing on terms and referents) as no target words repeat but rather only through participants learning to overcome egocentric bias. Our results, which suggest that this kind of improvement is very difficult, speak to a broader literature concerning the development of theory of mind and perspective-taking across the lifespan as well as ways to manipulate, train, and incentivize perspective-taking. There is considerable debate in the broader literature on theory of mind whether representing others’ mental states is automatic or requires effort (see e.g., [48–50]). Given the performance differences (both in terms of success and response time) between symmetric and asymmetric trials in our experiments, it seems unlikely that perspective-taking is automatic and effortless in our task. Beyond a certain age in childhood, what appears to improve about perspective-taking is not the *ability* to represent the mental states of others but rather the motivation and capacity to deploy it [22, 51–54]. The lack of improvement in our experiments may therefore also be related to individual differences in general executive functions. We did not measure these independently but note that the above-baseline performance suggested that participants were not prevented from taking perspective due to differences in executive functions. Nevertheless, future studies should take such factors into account. We believe our study shows that what improves with repeated interactions in real-life communication is not a (further) reduction of egocentric bias but rather effects of interacting about the same referents, giving person-specific feedback and asking for clarification, and adjusting. These were all absent from our experiments and do not strictly require perspective-taking.

It is possible that directors in Experiment 2 used frequency as a heuristic for narrowing down the search space (given the target word, think of associated rare words and select the one most likely to lead the matcher to the target word). Most of the optimal clue words that were given as part of the feedback after an incorrect trial in Experiments 2–3 were rare (words like ‘origami’, ‘eczema’, ‘retina’, and ‘bubonic’), which participants may have picked up on. Additionally, Zipf value changed across rounds in Experiment 2 but not in Experiments 1 or 3. The idea that the frequency heuristic helps because it limits the search space fits well with the findings from Sulik and Lupyan [29] where participants appeared to behave egocentrically when *generating* clues but were able to *evaluate* potential clues allocentrically when the clue space was limited. Perhaps there is no necessary strict dissociation between an improved perspective-taking account and a frequency-based heuristics account–discovering the frequency heuristic could limit the search space and thus allow for allocentric evaluation of potential clue words. Participants in Experiment 2 did not just start using random rarer clue words, the clue words they chose also had increasing allocentric salience, supporting the idea that they were able to evaluate the clue words in a non-egocentric way.

### 5.3 Limitations and alternative explanations

It is important to note that Experiment 3 (which featured the same rich feedback as Experiment 2) did not show the same effects as Experiment 2, posing the question: If participants discovered and used a frequency heuristic in Experiment 2, why was this heuristic not discovered in Experiment 3 where participants received the same feedback as in Experiment 2? One possibility, suggested by a reviewer, is that participants in Experiment 3 (recruited from Prolific) were not as highly educated as participants in Experiment 2 (recruited from a university setting) and thus had less access to infrequent clue words. While we do not have access to information about the level of education of the participants in Experiment 3, this idea is not supported by post-hoc analyses on the Zipf value of clue words. This analysis did not indicate that participants in Experiment 3 used significantly more frequent words than participants in Experiment 1 (as evaluated using a mixed effects linear regression on the combined data from Experiments 1 and 3, with clue Zipf value as the dependent variable and experiment as the predictor; Zipf values were not significantly higher in Experiment 3, b = 0.08, SE = 0.05, p = .090). Another potential explanation is that participants in Experiment 3 used this frequency heuristic strategy but only for the asymmetric accessible target words that most closely matched the targets in Experiment 2. However, post-hoc analyses indicated that this was not the case: neither success nor Zipf value improved for these asymmetric target words in Experiment 3. This suggests that the crucial feature leading to (modest) improvement over time in Experiment 2 was the consistency of the target words: all trials were asymmetric in Experiment 2, meaning that an egocentric approach would never lead to success, allowing participants to focus on honing their perspective-taking strategy (including, possibly, identifying a frequency-based shortcut). To tease apart the perspective-taking and heuristics explanations, future studies could ask the individual participants about their word associations and include these instead of the population-level word association norms used in the current study. Improvement over the course of the game would then mean that participants were adjusting to a specific partner’s perspective rather than employing general heuristics (such as ‘just use a related rare word’). In our experiments, participants appeared to find perspective-taking most difficult (or at least did not improve over time) when they had to alternate between symmetric and asymmetric trials. This may go some way towards explaining the conflicting results from some of the visually grounded perspective-taking tasks mentioned in the Introduction where in some study designs participants face the equivalent of a mix of symmetric and asymmetric trials, i.e., some occasions where their own perspective matches their partner’s, and some where it does not [12, 13, 20, 22].

A reviewer raised the concern that participants’ failure to show convincing improvement may be related to the recursive nature of perspective-taking. As we noted in Section 1.3. above, we adopted the simplifying assumption that inferences about one’s partner bottom out in a model of a simple interlocutor, i.e., one who relies on forward associations (e.g., [41]). The concern that was raised is that participants may be making successful inferences but making them at the wrong level of recursion, e.g., if the director not only simulated the matcher’s response to a specific clue but also modelled the matcher’s model of what prompted the director to generate that specific clue. While this is a valid concern, it seems unlikely that this was a widespread issue in our experiments given that we do not see forward association strength consistently dropping for either participant. In fact, none of our dependent variables show discernible patterns of either participant changing their behavior in the game (see Figs 6, 10, and 15). We would *not* be able to detect it if participants constantly oscillated between trying to take perspective and assuming that their partner was, consistently “missing” each other because they were making inferences at the wrong level of recursion. However, it seems highly unlikely that this would continue throughout the 120 trials.

## 6. Conclusion

We report results from three experiments in which participants played an open-ended guessing game, generating clues to communicate target words and generating guesses based on those clues. This task was challenging, but participants performed well above an egocentric baseline from the start, indicating that they were able to deploy perspective-taking from early on in the task. However, there was very little evidence that participants were able to improve performance over time; we only saw improvement over rounds in one of three experiments, and even there the improvements in performance were very modest and only on a subset of our different measures of perspective-taking. Furthermore, in experiments where they were available, participants found target words which afforded an egocentric approach (symmetric targets, where the high-ranking associates of the target word had the target word as their high-ranking associates) easier. We can conclude that perspective-taking is effortful and demanding, especially in circumstances without context where the search space for both signal and interpretation are unconstrained, and as a result improving perspective-taking over the course of a task is difficult.

The failure to improve in Experiments 1 and 3 was probably located at the search part of the task, as results from Experiment 2 indicated participants might be able to improve their performance very slightly under optimal circumstances when faced with a consistent perspective-taking task (i.e., no symmetric words) and given more guidance about the kinds of words they should be searching for.

In contrast to most previous studies, but following Sulik & Lupyan [29, 35], the present study examined the power of perspective-taking as a stand-alone mechanism without visual grounding. Our findings indicate that while perspective-taking might play a foundational role in ordinary communication, there are quite strong limits on people’s ability to adapt and improve perspective-taking without the context provided by interaction history and growing common ground.
